# Biotechnological and genetic innovations to enhance sorghum adaptation under climate change

**DOI:** 10.3389/fpls.2026.1757792

**Published:** 2026-02-23

**Authors:** Zhifang Wang, Jingzhen Wang, Ming Cheng, Yiming Du, Ian Godwin, Lingqiang Wang, Peng Lv, Guoquan Liu

**Affiliations:** 1 Institute of Millet Crops, Hebei Academy of Agriculture and Forestry Sciences and Hebei Branch of China National Sorghum Improvement Centre, Shijiazhuang, China; 2 State Key Laboratory of Conservation and Utilization of Subtropical Agricultural Biological Resources, Guangxi Key Laboratory of Sugarcane Biology, College of Agriculture, Guangxi University, Nanning, China; 3 College of Plant Science and Technology, Huazhong Agricultural University, Wuhan, China; 4 Plant Synthetic Biology Australia, School of Agriculture, Food and Wine, Adelaide University, Waite Campus, Glen Osmond, SA, Australia; 5 Hebei Youth Cadres Administrative College, Shijiazhuang, China; 6 Centre for Crop Science, Queensland Alliance for Agriculture and Food Innovation, The University of Queensland, St Lucia, QLD, Australia

**Keywords:** food security, climate change, sorghum, genetic engineering, genome editing, apomixis

## Abstract

Modern society is facing unprecedented global challenges, particularly climate change and food insecurity, which are intensifying the demand for crops capable of maintaining high yields under heat, drought, and salinity stress. Enhancing crop productivity and adaptation under climate change have thus become a global priority for agriculture research. Sorghum (*Sorghum bicolor* L. Moench), the fifth most important cereal crop worldwide, is increasingly recognized for its potential to strengthen food security, especially in arid and semi-arid regions. Its inherent tolerance to harsh environmental conditions makes it a promising candidate for sustainable agriculture. Recent biotechnological and genetic innovations in sorghum, including key gene discovery for agronomic traits, genotype-independent transformation using *WUS2* and *BBM*, RNA interference (RNAi) for improving grain quality, CRISPR-based and transgene-free genome editing, and emerging nanobiotechnologies, have been developed, applied and evolved to increase resistance to biotic and abiotic stresses, grain yield, biomass, and nutritional quality. Those innovations have enabled precise manipulation of sorghum’s genome, acceleration of breeding programs, and improvement of sorghum performance under environmental stress. Moreover, cutting-edge biotechnological and genetic innovations, such as nanobiotechnology, ultimate genotyping, and synthetic apomixis, have demonstrated immense potential for future sorghum development and improvement. Collectively, through integration of biotechnological and genetic innovations, the better sorghum lines can be developed with significantly enhanced adaptability, productivity, and nutritional value in the face of global climate challenges. This review highlights the pivotal role of innovation and provides a comprehensive overview of current research trends in sorghum to mitigate climate change, enhance adaptation, and strengthen global food security.

## 1 Introduction

**Figure 1:**
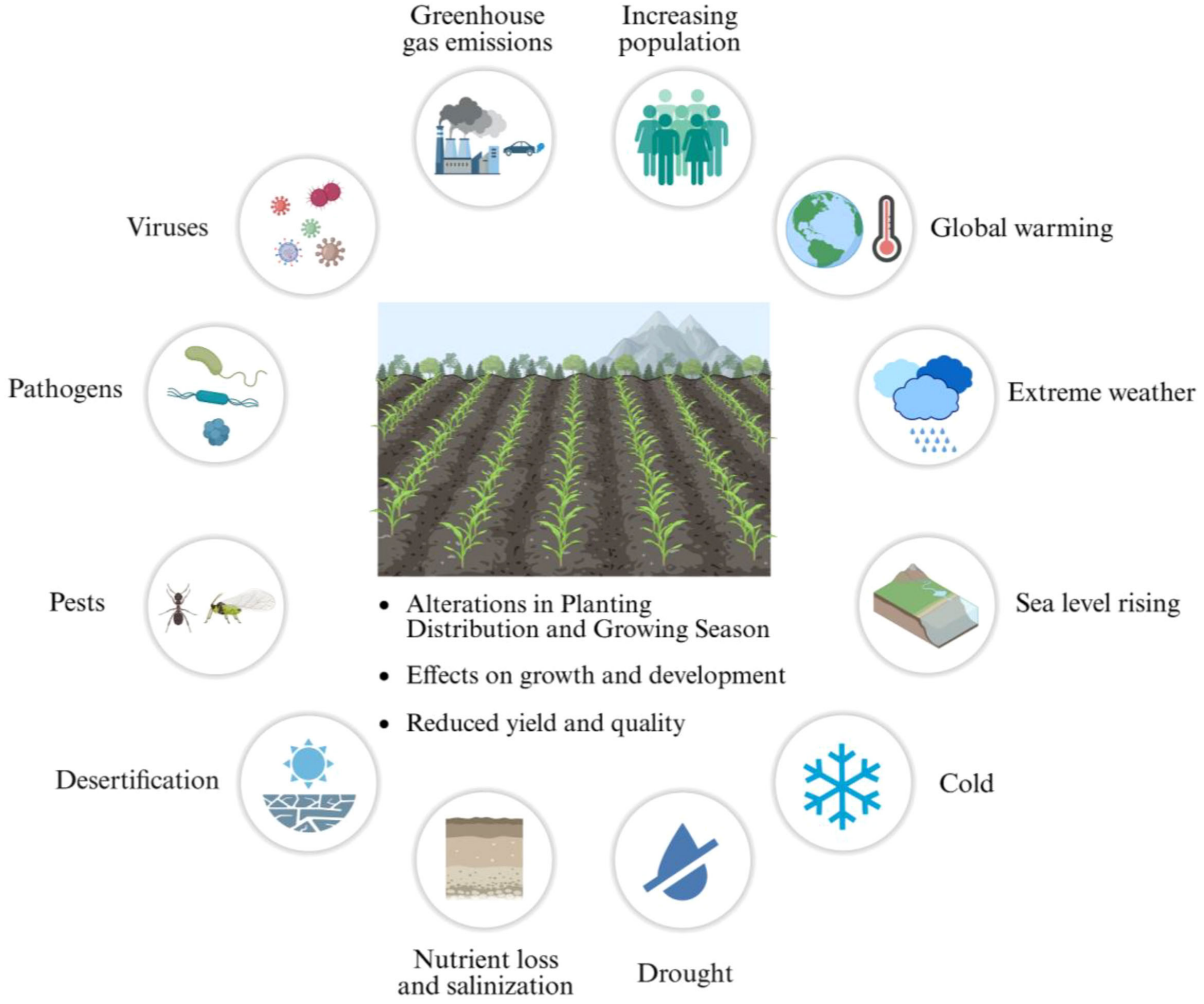
Global challenges on agriculture and food security. The rapid growth of the global population is not only one of the most pressing challenges affecting food security, but it also intensifies the demand for resources, bringing additional challenges to sustaining food production with the limited resources. Additionally, population growth imposes immense pressure on the ecological environment, indirectly impacting agricultural production. Climate change such as temperature shifts, droughts, water scarcity, and land degradation, along with the resulting biotic and abiotic stresses, further exacerbates agricultural pressure. These factors collectively contribute to the strain on global food supply, particularly in regions with fragile ecological environments. Created in BioRender. Wang (2025) https://BioRender.com/mdv7m7y.

Climate change has critically endangered global food security through intensifying impacts like erratic rainfall, temperature shifts, droughts, water scarcity, and land degradation according to the report from the intergovernmental panel on climate change (IPCC) (IPCC, 2023; **Figure 1**) (Musidzaramba et al., 2025). These extreme weather events have disrupted crop productivity, destabilized agriculture system, and worsened food availability worldwide (**Figure 1**) (Hultgren et al., 2025; Peng and Berry, 2019). Tropical regions, especially sub-Saharan Africa and South Asia, face acute risks as rural communities depend heavily on climate-vulnerable farming. The IPCC warns that global warming could drive over 122 million people into extreme poverty by 2030. By 2100, temperatures may rise 2 to 4.5 °C, with altered rainfall patterns, reduced monsoons, and erratic precipitation, further threatening food systems (Raza et al., 2019). Arid and semi-arid tropical (SAT) regions, covering 30% of land and 20% of the population globally, face disproportionate crop yield declines due to heat and drought. With the global population projected to hit 9.7 billion by 2050, urgent, tailored climate adaptation strategies for SAT regions are essential to mitigate hunger, poverty, and malnutrition (Chadalavada et al., 2021). Therefore, the demand for food has experienced a significant increase (**Figure 1**).

Cereal crops including wheat, maize, and rice are global staple foods. To meet food demands requires a 70-100% production increase by 2050, challenged by climate change, shrinking arable land, and droughts (Chadalavada et al., 2021; Godfray et al., 2010). Climate-resilient crops like sorghum and millet, which thrive in semi-arid regions and offer nutritional benefits, could sustainably replace traditional staples under intensifying climate change.

Sorghum (*Sorghum bicolor* L.) is one of the earliest cultivated cereals and ranks as the fifth globally after rice, maize, wheat, and barley. Originating in Africa, sorghum underwent gradual domestication during its transregional dissemination, with China emerging as one of the important domestication areas (Morris et al., 2013). It is widely recognized for its robust adaptability to harsh environments, including drought, flooding, saline-alkali conditions, and poor soil quality (Calviño and Messing, 2012; Xie and Xu, 2019). It can be cultivated in mountainous areas, saline-alkali lands, arid and semi-arid agroecosystems without competing for the limited arable land used for maize, wheat, or rice. Grown in over 100 countries, covering about 51.83 million hectares, primarily in Asia and Africa, it is one of the five major global cereal crops (Hariprasanna and Patil, 2015). It is consumed as a staple food for more than 500 million in developing countries like Africa and Asia, providing carbohydrates, proteins, and micronutrients. In developed nations such as Australia, it remains a crucial feed resource. Additionally, sweet sorghum and grain sorghum have gained significant attention from enterprises and farmers, with its cultivation area increasing annually internationally. Moreover, the nutritional value and diverse uses of sorghum highlight its agricultural significance, positioning it as a key component of food security (Bellasio et al., 2023; Pennisi, 2009).

Higher demands have been placed on basic sorghum research and breeding. Currently, the breeding of sorghum varieties that are widely adaptable, high-yielding, high-quality, strong in resistance, especially herbicide-resistant, is an essential goal for basic sorghum research and industrial application (Chadalavada et al., 2021; Endalamaw et al., 2025). Biotechnological and genetic innovation has demonstrated the massive impact on research and industrial application. For a long time, sorghum has been considered one of the most challenging crops for tissue culture, regeneration, gene delivery, and genetic transformation (Liu and Godwin, 2012a). As a result, the application of cutting-edge biotechnologies to enhance the genetics improvement of sorghum has lagged behind compared to rice and maize. However, significant progress has been made in last two decades.

As a C_4_-type model plant, it provides valuable inspiration for other C_4_ crops with complex genomes, including sugarcane. The development of biotechnologies and genetics, especially the advancement of sorghum genome T2T sequencing (Wang et al., 2025), genotype-independent genetic transformation (Fontanet-Manzaneque et al., 2024a; Lowe et al., 2016; Mookkan et al., 2017), transgene-free genome editing (Zhang et al., 2025b), the synthetic apomictic sorghum (Simon et al., 2025), and nanobiotechnology in sorghum (Yong et al., 2025), has greatly facilitated basic sorghum research and industrial applications in recent years.

This review provides an overview of the current state of sorghum biotechnological and genetic innovation and its strategies to enhance resistance, adaptation, and productivity. It reveals the tremendous potential to develop better sorghum through biotechnological and genetic innovations for mitigating the adverse effects of climate change and improving global food security, as well as the immense opportunities for future sorghum research and industrial applications.

## 2 The significant role of sorghum in global food security

### 2.1 Agronomic resilience of sorghum

**Figure 2:**
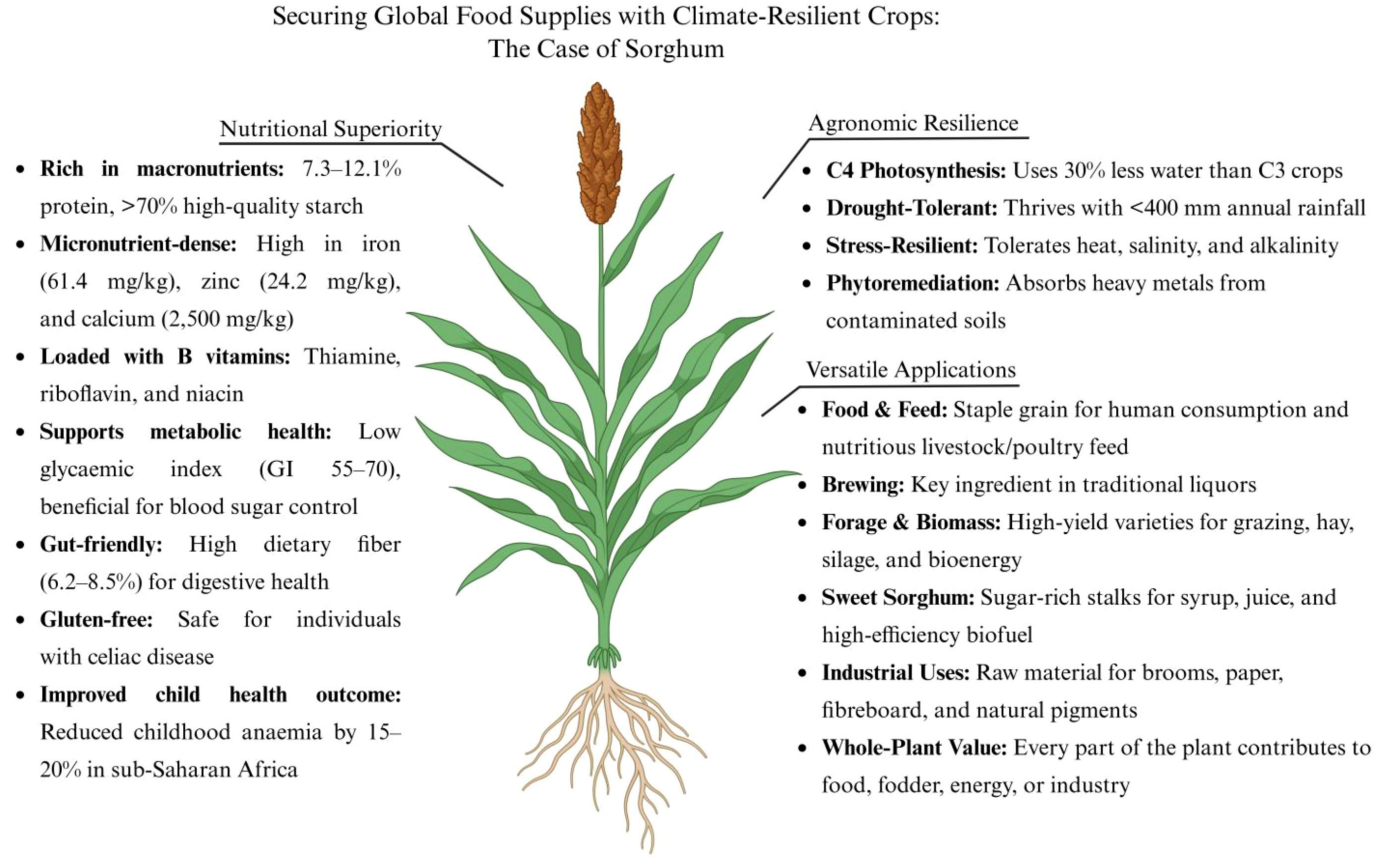
Sorghum: A climate-resilient crop for securing global food supplies. Created in BioRender. Cheng (2025) https://BioRender.com/woleuko.

Sorghum can grow on marginal lands due to its remarkable tolerance to various abiotic stresses such as drought, high salinity, and low nutrient levels (**Figure 2**) (Berenji et al., 2011; Maharajan et al., 2021; Woldesemayat and Ntwasa, 2018). Sorghum primarily grows in arid regions of Asia and Africa, known as the “camel of crops” for its ability to thrive in drought-stricken soils and endure prolonged droughts (Assefa et al., 2010; Hariprasanna and Patil, 2015). Several potential sources of stay-green characteristics have been identified in sorghum, contributing to yield under both well-watered and drought conditions (Borrell and Hammer, 2000; Jordan et al., 2012). As a C_4_ plant, Sorghum has over 30% greater water-use efficiency than C_3_ crops (FAO, 2020). This allows it to thrive in areas with less than 400 mm of annual rainfall, supporting farmers in resource-limited regions. Potential traits, associated with tolerance to heat stress, have been found in sorghum, such as early morning flowering and reduced canopy temperature. The early morning flowering trait allows sorghum plants to avoid or escape the high temperatures in the middle day. Furthermore, various sorghum genotypes demonstrate cooler canopies (escape) or higher canopy temperatures (tolerance), allowing them to either evade or endure excessive tissue temperatures while sustaining higher yields (Prasad et al., 2021). Additionally, sorghum displayed superior salinity-alkalinity tolerance compared to other grass family crops. Some sorghum varieties exhibit strong metal-absorption capabilities, showing potential for phytoremediation in soils contaminated with heavy metals (Mishra et al., 2021; Naseem et al., 2018).

The climate resilience of sorghum is shaped by an intricate interplay of morphological, physiological, and molecular mechanisms. Key traits contributing to this adaptability include a well-developed root system, precise regulation of stomatal activity, and the synthesis and accumulation of osmoprotectants (Enyew et al., 2025). These osmoprotectants, such as proline, glycine betaine, and sugars, are accumulated in plant cells to withstand abiotic stress conditions, including drought, salinity, and extreme temperatures (Enyew et al., 2025).

### 2.2 Versatile applications of sorghum

Sorghum boasts extensive utility across multiple sectors, including food, feed, brewing, and industrial processing. It is classified into four major types based on end-use: grain sorghum, forage sorghum, biomass sorghum, and sweet sorghum (Silva et al., 2022). Grain sorghum, grown primarily for its edible seeds, is a staple food and animal feed in many African and Asian regions. Incorporating sorghum grain into livestock and poultry feed prevents gastrointestinal diseases and improves meat quality. Grain sorghum is essential to China’s liquor production, with most of its output used for brewing, creating a unique liquor style (Meng et al., 2025). Forage and biomass sorghum varieties are valued for their high biomass yield, making them ideal for animal feed through grazing, hay, silage, and bioenergy production (Moore et al., 2020). Sweet sorghum, known for its sugar-rich stalks (crushable for juice extraction and processing), is highly valuable for syrup and biofuel production (Reddy et al., 2005) and has the highest alcohol conversion rate among crops, indicating its potential to impact renewable energy production worldwide (Hu et al., 2022). Sorghum has various industrial applications, including brooms, paper, fibreboard, and natural red pigments. Every part of the plant holds economic value, showcasing its potential for development and commercialization. Thus, sorghum has become essential for food, fodder, energy, and processing industries (**Figure 2**).

#### 2.3 Nutritional superiority of sorghum

Sorghum grain is recognized as a nutritionally dense staple crop. In arid regions of Asia and Africa, sorghum has become a key crop for food security and nutrition due to its resilience and nutrient profile (de Oliveira and de Alencar Figueiredo, 2024). It is nutritionally recognized as a “nutrient treasure trove”. Protein content is 7.3% to 12.1%, with high-quality starch over 70%. Notably, its exceptional mineral profile includes iron (61.41 mg/kg) and zinc (24.23 mg/kg), both at levels significantly higher than those found in other cereal crops. Sorghum contains up to 2,500 mg/kg of calcium, 5 to 7 times more than common grains. It is rich in B vitamins like thiamine, riboflavin, and niacin, along with various antioxidants (Shegro et al., 2012). Sorghum offers unique health benefits: its low glycaemic index (GI 55-70) helps blood sugar control for diabetics, its high dietary fibre (6.2-8.5%) enhances gut health, and its gluten-free nature makes it ideal for those with celiac disease (de Oliveira and de Alencar Figueiredo, 2024; Kumar et al., 2018). While antinutritional factors like phytic acid may reduce mineral bioavailability, traditional methods such as fermentation and sprouting can enhance iron and zinc absorption by 40-60%. In sub-Saharan Africa, adopting sorghum has decreased childhood iron-deficiency anaemia by 15-20% in about 2 million children (**Figure 2**) (de Oliveira and de Alencar Figueiredo, 2024).

## 3 Biotechnological innovations in sorghum

**Figure 3:**
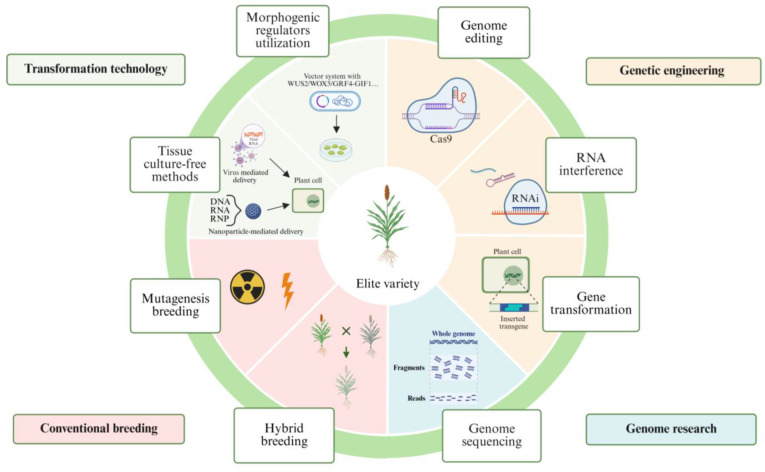
Biotechnological and genetic innovations in sorghum. Conventional breeding, such as hybridization and mutation breeding approaches, serves as the foundational method for sorghum improvement but is limited by its time-consuming and labor-intensive. Sorghum genetic engineering including gene transformation, genome editing, and RNA interference (RNAi) enables the direct development of desired traits through genetic modification, offering valuable insights for crop improvement. With significant advancements in sorghum genome research, the exploration and accumulation of key functional genes in sorghum have become more convenient, which has accumulated essential genetic resources for sorghum genetic engineering breeding. Moreover, the development of sorghum genetic transformation technologies has provided critical support for the widespread application of genetic engineering techniques in sorghum. In particular, the use of morphogenic regulators has enabled genetic transformation in sorghum to overcome genotype limitations and achieve higher transformation efficiency. Furthermore, continuous exploration and research in sorghum delivery methods have led to the development of approaches that do not rely on tissue culture, such as virus- or nanoparticle-mediated delivery methods. These advances have significantly improved sorghum genetic engineering technologies and established an important foundation for the rapid creation of new sorghum varieties in the future. Created in BioRender. Wang (2025) https://BioRender.com/cz9o6mo.

Genetic transformation is a vital tool for precise manipulation of sorghum traits. The traditional tissue culture process includes callus induction, proliferation, regeneration, maturation, and rooting. Due to strong genotype dependency, the callus proliferation stage is often the limiting step. Successful transformations are mainly limited to genotypes like Tx430 and P898012, hindering genetic improvement in others (Liu and Godwin, 2012b; Wu et al., 2014). Sorghum stable transformation efficiency was consistently below 10% before 2010, but optimization of tissue culture medium and biolistic bombardment parameters boosted the efficiency of sorghum Tx430 to 20.7% (Liu and Godwin, 2012b). In subsequent studies, although the genetic transformation efficiency of sorghum Tx430 reached a breakthrough of 46.6%, the genotype limitation was still not overcome—until morphogenic regulators were applied to sorghum genetic transformation (Belide et al., 2017; Lowe et al., 2016). Compared with Tx430, the transformation efficiency of the recalcitrant sorghum cultivar P898012 remained relatively low, not exceeding 10%, even with the application of morphogenic regulators (Mookkan et al., 2017). Since enhancing sorghum genetic transformation involves two main approaches: introducing morphogenic regulators and optimizing parameters, therefore, the transformation methods and parameters are continuously optimized (**Figure 3**) (Che et al., 2022; Fontanet-Manzaneque et al., 2024a; Li et al., 2024b; Lowe et al., 2016).

### 3.1 Transcription factors enhance plant regeneration and transformation.

Several key genes and transcription factors have been identified to enhance monocot plant transformation and regeneration efficiency. Notable examples include *WUSCHEL* (*WUS*), *BABY BOOM* (*BBM*), *WOX* family members, *AGAMOUS-Like 15* (*AGL15*), and the wound-induced dedifferentiation gene *WIN* (Nigam et al., 2025). Other promising morphogenic regulators include *LEAFY COTYLEDON 1* (*LEC1*), *LEC2*, *MONOPTEROS* (*MP*), *SHOOT MERISTEMLESS* (*STM*), and *ISOPENTENYL TRANSFERASE* (*IPT*). Using these factors together can enhance transformation efficiency (Silva et al., 2022). *Agrobacterium*-mediated transformation of the *WUSCHEL 2* (*WUS2*) gene induces direct somatic embryo formation and regeneration, bypassing genotype-dependent callus formation and shortening the tissue culture cycle (Che et al., 2022; Wang et al., 2023). This method enhances regeneration capacity and transformation efficiency in sorghum. Combined with advanced gene excision systems, such as Cre-LoxP system, it enables high-quality transformation events free of morphogenic genes and selectable markers (Che et al., 2022). Notably, WUS2-assisted transformation bypass genotype-dependent callus formation and significantly shorten the duration of tissue culture achieves a 6.8-fold increase in CRISPR/Cas9-mediated gene knockout efficiency across multiple target sites in various sorghum genotypes (Che et al., 2022). In sorghum, non-integrative *BABY BOOM* and *WUS2* genes overcome the limitations of *Agrobacterium* transformation based on immature embryo-derived calli of the BTx430 genotype. This method cuts the transformation cycle by 40%, boosts independent transformation events per immature embryo, and offers a breakthrough for sorghum functional genomics and precision breeding (Nelson-Vasilchik et al., 2022). Furthermore, the application of novel promoter combinations to regulate the expression of *Wus2* and *Bbm* promotes the rapid formation of somatic embryos and the regeneration of T_0_ plants from seedling-derived early leaf tissues of maize and sorghum after Agrobacterium infection, while this improved leaf transformation method enables Cas9-mediated genome modification in multiple gramineous species including maize and sorghum (Wang et al., 2023b). However, constitutive overexpression of *BBM* and *WUS2* can harm plant development, causing leaf distortion and reduced fertility. Researchers typically remove the *BBM* and *WUS2* expression cassettes before shoot regeneration or use altruistic morphogene-assisted transformation (MAT) to address these issues. These methods increase workload and decrease transformation efficiency due to incomplete removal or insertion of developmental helper genes (Aregawi et al., 2022). It was shown that chimeric sequences of GRF transcription factors and their GIF cofactors significantly improve regeneration efficiency in both monocot and dicot species, expanding transformable varieties and producing fertile transgenic plants. Notably, the GRF4-GIF1 chimera promotes embryogenesis and shoot proliferation in wheat without requiring extra cytokinin supplementation (Debernardi et al., 2020). It was demonstrated that GRF4-GIF1 and GRF5 enhance sorghum transformation efficiency, reducing the process to under two months—an improvement not seen with BBM-WUS systems (Li et al., 2024b). The combination of GRF4-GIF1 and the helper plasmid pVS1-VIR2 achieved the highest transformation efficiency at 38.28%, a 7.71-fold increase, while overcoming growth defects associated with BBM-WUS. Crucially, the CRISPR/Cas9 gene editing tool, developed from the GRF4-GIF1/ternary vector system achieved a 41.36% gene mutation efficiency in sorghum, successfully creating null mutants and offering a stable solution for precision breeding (Li et al., 2024b). Recently, a novel regeneration regulator *ZmHSCF1*, which promote embryogenic callus formation and proliferation, has been identified. The innovation can be utilized for improving genetic transformation and accelerate crop improvement (**Figure 3**) (Li et al., 2025). As key regulators of cell fate, morphogenic factors are playing crucial roles in surmounting genotypic restrictions and markedly boosting genetic transformation efficiency in monocots via the induction of somatic embryogenesis and the promotion of recipient cell dedifferentiation.

### 3.2 Tissue culture-free methods are promising

#### 3.2.1 Virus-induced gene silencing (VIGS) offers a powerful tool for functional genomics

With the advancement of plant molecular biology and genome editing technologies, scientists have discovered that plant RNA viruses are highly efficient at delivering gene-editing components into plant cells. These viruses possess features including well-organized structures, efficient replication mechanisms, high-level transient expression without the integration of exogenous DNA into the host genome, and the ability to spread systemically in plants, which make them ideal vector candidates. In Cas9-transgenic plants, virus-mediated sgRNA delivery can produce heritable edits, while virus-induced gene silencing (VIGS) offers a powerful tool for functional genomics. Although sorghum is generally recalcitrant to Brome mosaic virus (BMV) infection and thus unsuitable for VIGS studies, Singh et al. (2018) successfully established an efficient BMV-based VIGS system. By rub-inoculating young sorghum leaves with sap extracted from BMV-infected *Nicotiana benthamiana* leaves, gene silencing was monitored in sorghum inflorescences (Singh et al., 2018). The Foxtail mosaic virus (FoMV)-based system outperforms barley stripe mosaic virus (BSMV), and has shown exceptional performance in sorghum, achieving up to 60% somatic mutation frequency with apparent phenotypic effects. By leveraging a replicating coat protein promoter strategy, FoMV successfully drives sgRNA expression and has edited multiple key genes, confirming its potential for monocot gene editing (Baysal et al., 2025). Butler et al. (2025) demonstrated that combining morphogenic regulators with FoMV and conventional T-DNA vectors enables genetic transformation using sorghum leaf tissues as explants, which can generate transgenic embryogenic calli and shoots (Butler et al., 2025). In the future, optimizing viral vector properties could allow researchers to more efficiently generate heritable DNA-free genome-edited plants, simplify the tissue culture process, expand their applicability across various crops, and create transformative opportunities for agricultural improvement (Ma et al., 2020) (**Figure 3**).

#### 3.2.2 Nanoparticle-mediated delivery of nucleic acids and proteins into plants

Virus-mediated gene delivery has been successful in some plant species; however, its host range is also restrictive. There is a need to develop safer, more reliable, and universal platforms for delivering functional biomolecules into intact plants for a wide range of applications in plant biotechnology (Ali et al., 2022).

Nanoparticle-mediated delivery of biomolecules has already had an immense impact in the biomedical field (Cunningham et al., 2018; Yong et al., 2025). Owing to their ability to penetrate plant cell walls without external force, nanoparticles are widely applicable to diverse plant species. In addition, nanomaterials can protect cargos (such as DNA, RNA, RNP et al.) from degradation and reach previously inaccessible plant tissues, cells, and subcellular locations. All these properties render nanoparticles ideal materials for the delivery of exogenous biomolecules. Although delivering biomolecules into plants faces the additional challenge of the plant cell wall, several recent studies have demonstrated the nanoparticle-mediated delivery of functional plasmid DNA, dsRNA, or siRNA into plants (Yong et al., 2025). Layered double hydroxide (LDH) nanoparticles are a family of clay materials with considerable potential as a low-cost, non-toxic vector system for delivering biomolecules in plants (Yong et al., 2025). Most reported studies focus on leaf tissue, with recent increases in reports about the nanoparticle delivery of functional nucleic acids into roots (Demirer et al., 2019; Lew et al., 2020). Interestingly, plant roots can absorb protein molecules from plant culture media (Yong et al., 2025), and protein complexes on the plant cell membrane can be internalized through the active process of endocytosis (Cunningham et al., 2018; Lew et al., 2020). A recent study demonstrated that LDH nanosheets coated with lysozyme are actively taken up into the root tip, root hairs, and lateral root junctions by endocytosis, and translocate via active membrane trafficking, using seedlings from plants *N. benthamiana*, *Arabidopsis*, tomato, and sorghum (Yong et al., 2025). Because it is not restricted by genotype, easy to manipulate, and elimination of tissue culture requirements, this approach possesses substantial potential to improve recalcitrant, genotype-constrained crops, which in turn shortens the breeding cycle (Yan et al., 2023; Yong et al., 2025).

## 4 Application of biotechnological innovations in sorghum

Over the past four decades, sorghum biotechnological innovation has advanced from basic transformation techniques to a refined precision breeding system. Initial work focused on introducing foreign genes into sorghum protoplasts, followed by significant achievements in the stable production of transgenic sorghum using particle bombardment and *Agrobacterium*-mediated transformation (Casas et al., 1993; Ou-Lee et al., 1986; Zhao et al., 2000). Subsequent advancements, including optimized *Agrobacterium* and Biolistic bombardment-mediated protocols, have significantly improved genetic transformation efficiency. To precisely engineer sorghum traits, it is often necessary to overexpress, or silence, or modify gene function in a targeted manner (Lamont et al., 2017).

### 4.1 Overexpression of the genes in sorghum

Compared to conventional breeding, gene overexpression technology offers precise regulation of target gene expression, cross-species introduction of superior genes, and rapid trait improvement, providing powerful technical support for crop genetic enhancement. In addition to the highly efficient genetic transformation methods mentioned above, promoter selection is critical for the precise regulation of gene expression. Constitutive promoters drive gene expression in all tissues, while tissue-specific and inducible promoters selectively regulate expression in specific tissues, developmental stages, or under stress conditions. Constitutive promoters, such as CaMV 35S, are common in dicots but exhibit unstable expression in monocots like sorghum. In contrast, the maize ubi-1 promoter exhibits 10-fold higher expression in monocots, making it a more suitable option (Christensen et al., 1992).

The core function of a tissue-specific promoter is to drive the precise expression of exogenous genes exclusively in specific tissues or organs of plants, while restricting their transcription in non-target tissues. This enables the targeted improvement of transgenic traits and the avoidance of potential adverse effects. Thus, tissue-specific promoters, such as the endosperm-specific *α*-kafirin and *β*-kafirin promoters, or the developmentally regulated *Sh2* promoter, are increasingly valuable (Liu et al., 2020). The stem-specific *A2*/*LSG* promoter drove the expression of vacuole-targeted *SUCROSE ISOMERASE* (*SI*), achieving a breakthrough in sugar accumulation. In T_0_ transgenic grain sorghum, stems accumulated 50–60% isomaltulose, with total sugar content reaching 1000 mM which is equivalent to an 8-fold increase over controls (118 mM). When elite engineered lines (A5, LSG9) were crossed with sweet sorghum, F_1_/F_2_ hybrids exhibited >750 mM total sugar, surpassing conventional sweet sorghum (480 mM) and even field-grown sugarcane (600–700 mM) (Liu et al., 2021a). These promoter resources provide essential tools for the precision breeding of sorghum. Future research should focus on optimizing their applications to enable safer, more efficient transgenic crop development.

### 4.2 RNA interference (RNAi)

RNA interference (RNAi) is a powerful gene-silencing mechanism that functions by degrading messenger RNA (mRNA) prior to its translation. In recent years, the RNAi mechanism has emerged as a key gene-silencing tool and been widely applied in the field of plant functional genomics, owing to its conservation across diverse organisms and capacity for sequence-specific gene targeting. For example, Kafirin co-suppression is fundamental to improving sorghum protein digestibility and nutritional value (Elkonin et al., 2021). The nutritional quality of sorghum grain is constrained by a low content of essential amino acids and the protease resistance of its seed storage proteins (kafirins). Targeted RNAi-mediated suppression of specific kafirin subclasses, particularly the γ- and α-types, can effectively improve protein digestibility in sorghum while without compromising critical agronomic traits (Elkonin et al., 2021). RNAi suppression of the opaque2 gene in sorghum embryos lowered kafirin content significantly compared to wild type (Cai et al., 2019). In addition, the transgenic sorghums, which were developed using RNA interference (RNAi) to downregulate genes affecting grain size and protein body structure, had significantly higher crude protein (CP) and higher digestibility, demonstrating the commercial value through genetic engineering (Macelline et al., 2024). Moreover, RNAi technology shows promise for enhancing sorghum biomass. Suppressing *4CL*, a key gene in lignin biosynthesis, cut stem lignin by 25% and boosted cellulose and soluble sugar to 36.56% and 59.72%, respectively, significantly improving processing characteristics for forage sorghum (Bhanupriya and Kar, 2025). With the discovery of various classes of regulatory non-coding RNAs (e.g., miRNAs, phasiRNAs, and NAT-siRNAs), the range of RNAi applications has been expanded, which facilitate accurate post-transcriptional and epigenetic modulation (Chaudhary et al., 2024). These achievements provide technical support for sorghum quality improvement. In the future, RNAi will remain a vital component of the functional genomics toolkit and a key complement to gene-editing technologies. We should continue to refine our understanding of RNAi mechanisms, address issues related to targeting effects and long-term stability, improve its precision, expand its scope of application. Fully exploit the enormous potential of RNAi technology in crop genetics.

### 4.3 Genome editing and CRISPR/Cas systems

Gene editing technologies enable precise modifications at the DNA level, including targeted insertions, deletions, or base substitutions (Khalil, 2020). There are three major gene editing platforms: zinc-finger nucleases (ZFNs), transcription activator-like effector nucleases (TALENs), and the clustered regularly interspaced short palindromic repeats/CRISPR-associated proteins (CRISPR/Cas) system. ZFNs and TALENs were pioneers, but their complexity, high costs, and limited efficiency have led to the more versatile and user-friendly CRISPR/Cas system (Metje-Sprink et al., 2019). The CRISPR/Cas system has been implemented in over 50 plant species. It offers distinct advantages: simplified design protocols, reduced costs, and higher efficiency, making it an indispensable tool for precision breeding in sorghum. Multiple software tools, such as CRISPR-Pv 2, CRISPOR, CRISPRdirect, and CRISPR-PLANT, can design optimal targeting strategies for sorghum genome editing (Liu et al., 2020).

CRISPR/Cas9 editing of the *α*-kafirin-encoding *k1C* gene family in sorghum reduced *α*-kafirin content in seeds, enhancing protein digestibility and lysine levels. The study demonstrated that a single sgRNA effectively edited multiple genes despite mismatches, enabling non-transgenic, nutrient-rich sorghum development (Li et al., 2018). Sorghum in sub-Saharan Africa (SSA) faces severe threats from the parasitic weed *Striga hermonthica*. *LOW GERMINATION STIMULANT 1* (*LGS1*) is the only known gene locus influencing *Striga* resistance; loss-of-function alleles (*lgs1*) reduce *Striga* germination-stimulating activity. PCR markers detected lgs1 alleles in 6% of 406 sorghum accessions, identifying mutations causing gene loss, including three known deletions (*lgs1–1* to *lgs1-3*) and a novel 50-kbp deletion (*lgs1-6*) (Adeyanju et al., 2024). CRISPR/Cas9 edited strigolactone (SL) biosynthesis genes (*CCD7*, *CCD8*, *MAX1*) and a DUF gene in the *lgs1* region of two sorghum cultivars, achieving 70% transformation and 17.5% editing efficiency. Edited lines exhibited downregulation of SL pathway genes, reduced SL levels in root exudates, delayed Striga infection, and lower infestation rates while maintaining normal growth (Kaniganti et al., 2025). However, suppressing *Striga* germination by reducing SL biosynthesis leads to abnormal development in sorghum (Chen et al., 2018). Therefore, researchers shifted their focus to limiting SL secretion into the rhizosphere to suppress *Striga* germination. Shi et al. (2025) identified two *ABCG* transporter genes crucial for SL secretion to the sorghum rhizosphere. The individual or combined knockout of *SbSLT1* and *SbSLT2* could significantly impair *Striga* germination by disrupting SL export. These knockout lines exhibited lower levels of *Striga* infestation, leading to higher sorghum grain yields and biomass production (Shi et al., 2025). Temporal regulation is crucial for the diverse applications of sorghum cultivated globally. Char et al. (2019) developed a CRISPR/Cas9 system to edit the flowering-time gene *SbFT* (*Sb10G045100*) and gibberellin metabolism gene *SbGA2ox5* (*Sb09G230800*) (Char et al., 2019). Persistent Cas9/sgRNA activity induced novel site-specific mutations in progeny, with *SbFT* mutants showing significant flowering-time variations. Identifying and utilizing male sterility genes is essential for hybrid breeding. CRISPR/Cas9 knockout confirmed that the key male sterility gene, *MS8* (*Sobic.004G270900*), which encodes a conserved bHLH transcription factor, induced male sterility across genetic backgrounds (P<0.01) (Jiang et al., 2021). In China, sorghum brewing value is particularly prominent; “Moutai liquor” and traditional vinegar rely on it. By knocking out *SbBADH2* via CRISPR/Cas9, researchers overcame industrial limitations of the aromatic Indian cultivar IS19912, creating new germplasms with aromatic seeds and leaves. Animal trials confirmed improved leaf palatability (Zhang et al., 2022a). Furthermore, Cheng et al. (2024) successfully increased the content of 2-acetyl-1-pyrroline (2-AP) in sweet sorghum via targeted mutagenesis of the *SbBADH2* gene; concurrently, they achieved a breakthrough by establishing an Agrobacterium-mediated genetic transformation system and a CRISPR/Cas9-based genome editing system in “Gaoliangzhe” (GZ), an elite sweet sorghum accession, which laid a solid foundation for functional genomic research and biotechnological breeding of sweet sorghum cultivars (Cheng et al., 2024). Recently, Simon et al. (2025) first reported artificially induced apomixis in sorghum, demonstrating that asexual hybrid seeds derived from this approach stably transmit heterosis across generations (Simon et al., 2025). This work is a significant innovation, exemplifying the integrated application of sorghum genetic transformation and genome editing technologies; notably, it holds the potential to capture heterosis and preserve hybrid vigor via sorghum seeds, driving substantial agricultural advancements. Currently, enhanced fertility is a prerequisite for commercial grain production, and further refinements are needed to unlock the full agronomic potential of artificially induced apomictic sorghum in field settings (Simon et al., 2025).

Current genome editing in sorghum mainly focuses on gene knockout, but precision tools like base editing and prime editing show greater promise for accurate mutations. Researchers have created cytidine base editors (CBEs), adenine base editors (ABEs), and dual base editors capable of inducing both substitutions. Examples of these dual systems include SPACE, Target-ACEmax, STEME, A&C-Bemax, AGBE, and ACBE (Chen et al., 2024; Gaudelli et al., 2017; Grünewald et al., 2020; Komor et al., 2017; Li et al., 2020; Liang et al., 2022; Sakata et al., 2020; Zhang et al., 2024d, Zhang et al., 2020). Beyond base editing, promoter editing can also establish effective quantitative trait variations. Zhou et al. (2023) developed a computational model that assigns values to different promoter regions and created a CRISPR-Cas12a-based promoter editing system to guide promoter editing experiments for fine-tuning gene expression and generating desired quantitative traits (Zhou et al., 2023). Recently, a transgene-free genome editing system was established in sorghum through targeting *PHYTOENE DESATURASE* (*PDS*) gene to generate a visible phenotype mutation albino (Zhang et al., 2025b). Field-grown transgenic sorghum exhibits a high risk of unintended hybridization with closely related weedy relatives (e.g., Sorghum bicolor subsp. drummondii, commonly known as Sudan grass), which may result in the spread of transgenes and subsequent transgenic contamination in agricultural ecosystems (Wolfenbarger and Phifer, 2000). In sharp contrast, precision genome-edited sorghum lines can be generated without the integration of exogenous transgenes (He et al., 2022; Li et al., 2024a; Zhang et al., 2025b), thus circumventing the biosafety concerns associated with transgenic flow and hybridization. The production of transgene-free, site-specific edited sorghum germplasm therefore represents a critical strategy for mitigating the risks of transgenic contamination while retaining the agronomic benefits conferred by targeted genetic modifications. Given its robust editing efficiency, this system holds significant promise for future applications in sorghum improvement.

## 5 Genetic innovations in sorghum

Genomic innovations have formed the foundation of modern sorghum breeding and functional genomics. Key innovations in sorghum genomics, including the first reference whole genome sequencing, telomere-to-telomere (T2T) sequencing, pan-genome construction, and multi-omics databases. These resources have enabled the identification of structural variants, gene regulatory networks, and trait-associated loci, thereby facilitating high-resolution mapping and targeted breeding strategies. The integration of these genomic platforms is transforming sorghum research by unlocking its genetic diversity and enhancing its adaptability to climate stressors (**Table 1**).

**Table 1:** Genetic innovations in sorghum.

Research domain	Key resources/tools	Technical innovations	Major findings	Applications	References
Reference Genomes	• grain sorghum BTx623 (v1) (730Mb)	• Whole Genome Shotgun, WGS	• 98% genes anchored	Foundation for molecular breeding and genetic variation analysis	(Paterson et al., 2009)
	• grain sorghum BTx623 (v3.1.1)	• deep sequencing, genetic linkage analysis, and transcriptome data	• Enhanced sequence order, added 29.6 Mbp novel sequence, 24% more genes annotated (total 34,211), increased average gene length & N50, 10-fold lower error rate (1/100 kbp)		(McCormick et al., 2018)
	• grain sorghum Tx430	• Oxford Nanopore sequences generated on a MinION sequencer are combined with Bionano Genomics Direct Label and Stain (DLS) optical maps	• a scaffold N50 of 33.28 Mbps and covers 90% of the expected genome length		(Deschamps et al., 2018)
	• sweet sorghum cultivar Rio (729.4 Mb)	• PacBio RS II system + SMRT cell	• Key regulatory variations and deleterious mutations in sugar metabolism genes drive stem sugar accumulation		(Cooper et al., 2019)
T2T Genomes	• BTx623-T2T	• PacBio HiFi + UL-ONT + Hi-C	• Closed 3,913 gaps and corrected 1,131 misassemblies	• Enhanced GWAS resolution	(Deng et al., 2024)
	• Hongyingzi		• Revealed tannin pathway genes	• Baijiu sorghum breeding	(Ding et al., 2024)
	• Hongyingzi and Huandiaonuo		• Chinese Baijiu-brewing Sorghum T2T Genome Database (http://sorghum.org.cn/)	• First database for brewing sorghum featuring genome browsing	(Bao et al., 2024)
	• E048		• Discovered 2.9 Mb E048-specific region	• Disease resistance improvement	(Chen et al., 2025)
Pan-Genomes	• 13 varieties, including S. propinquum, wild sorghum, and cultivated sorghum	• Multi-omics integration	• The pan-genome spans 954.8 Mb, exceeding the reference genome (BTx623, 732.2 Mb) by 30%	• Reveals genetic basis of grain color via GWAS	(Tao et al., 2021b)
	• 354-accession	• Combined reference genomes with 354 population-scale sequencing data	• 79 drought genes missing in BTx623	• Drought adaptation breeding	(Ruperao et al., 2021)
	• 1,661-germplasm	• GWAS with structural variants	• Identified Dw3 height-control variants	• Solving height reversion	(Marla et al., 2025)
Multi-Omics Databases	• SorghumFDB (http://structuralbiology.cau.edu.cn/sorghum/)	• RNA-seq + ionomics	• GRN hubs (SbFIT/SbPYE)	• Functional genomics data mining platform with comprehensive gene annotations	(Tian et al., 2016)
	• SorGSD (https://ngdc.cncb.ac.cn/sorgsd/)	• EMS mutant libraries	• Nutrient stress networks	• MutMap-based gene cloning	(Liu et al., 2021b)
	• Chinese Baijiu-brewing Sorghum T2T Genome Database (http://sorghum.org.cn/)	• PacBio HiFi + UL-ONT + Hi-C	• HYZ and HDN share 7,264 unique gene clusters potentially involved in Baijiu flavor formation	• First database for brewing sorghum featuring genome browsing	(Bao et al., 2024)
	• SGMD (https://sorghum.genetics.ac.cn/SGMD)	• Integrated analysis pipelines	• 13,226 M1 mutants	• Multi-omics data mining	(Chen et al., 2025)
	• HEMU (https://shijunpenglab.com/HEMUdb/)	• 4,718 RNA-seq datasets	• 20 Andropogoneae species omics data	• Cross-species comparative analysis	(Zhu et al., 2024)
	• NEEDLE (https://github.com/DaeKwan-Ko/needle)	• Metabolomics-metatranscriptomics integration	• IAA/GA4-producing microbes	• Co-expression network analysis tool for non-model crops	(Ko and Brandizzi, 2025)

### 5.1 The sorghum reference genome is the blueprint for gene mining

Genomic analysis offers a fundamental blueprint of genomic information, serving as the foundation for molecular breeding. Genomic analysis of cereals enables the screening of wild and domesticated germplasms to identify novel sources of desirable traits. The first sorghum reference genome (from grain sorghum BTx623) was sequenced in 2009, with approximately 98% of genes anchored to chromosomes (**Table 1**) (Paterson et al., 2009). An improved version (BTx623 v3.1.1) was released in 2018 (McCormick et al., 2018). Subsequently, a chromosome-scale *de novo* assembly of the repeat-rich Tx430 genome was achieved by integrating Oxford Nanopore MinION sequencing data with Bionano Genomics Direct Label and Stain (DLS) optical maps in 2018 (Deschamps et al., 2018). Additionally, a high-quality reference genome for the sweet sorghum cultivar “Rio” was completed using Pacific Biosciences long-read sequencing technology in 2019 (**Table 1**) (Cooper et al., 2019). These genomic resources have been widely utilized to identify various genetic variations, including single-nucleotide polymorphisms (SNPs), insertions/deletions (InDels), structural variations (SVs), presence/absence variations (PAVs), and copy number variations (CNVs).

### 5.2 T2T no-gap genome is setting a new “gold standard” for genotyping

The complete telomere-to-telomere (T2T) assemblies enhance our understanding of genome structure, biology, and agricultural applications. Advancements in sequencing technologies, like PacBio HiFi (base accuracy >99.9%), ultra-long Oxford Nanopore Technology (UL-ONT; >100 kb), and Hi-C, have made T2T genome assemblies possible. Researchers assembled the BTx623 genome using Hifiasm with ultra-long ONT data (~82.2x coverage) and PacBio HiFi data (110.7x), then polished and corrected contigs using Hi-C (127.2x) and Illumina sequencing (65.4x), achieving a complete T2T assembly of all 10 chromosomes, including telomeres and centromeres (BTx623-T2T) (**Table 1**) (Deng et al., 2024). The BTx623-T2T genome covers 719 Mb, incorporating 43.6 Mb of new sequences compared to the previously published BTx623-v3.1, mainly in complex regions such as centromeres, telomeres, and other repetitive areas. Additionally, BTx623-T2T closed 3,913 gaps and corrected 1,131 misassemblies in BTx623-v3.1. Using transcriptome data from 76 multi-tissue/stage samples, 3,565 new protein-coding genes were annotated. The BTx623-T2T reference genome significantly improved the utilization of Illumina reads and alignment accuracy compared to BTx623-v3.1. A GWAS study on stem water content in 202 sorghum accessions found that using the BTx623-T2T reference genome identified all significant loci from BTx623-v3.1 and revealed three additional loci, highlighting its effectiveness for candidate gene discovery in agronomic traits (**Table 1**) (Deng et al., 2024).

The Hongyingzi (HYZ) variety, developed in 2008, has high grain tannin content and other desirable traits for distilled liquor (Baijiu) production, accounting for over one-third of China’s sorghum cultivation (Zhang et al., 2022b). Ding et al. performed a high-accuracy T2T assembly of the HYZ genome using UL-ONT, PacBio HiFi, and Hi-C. The complete *de novo* HYZ genome assembly fills gaps in tannin synthesis pathways and identifies genetic targets for Baijiu-focused breeding. Subsequently, Bao et al. released the Sorghum T2T Genome Database (http://sorghum.org.cn/), featuring high-quality T2T assemblies of two Chinese Baijiu landraces: Hongyingzi (used for Maotai, China’s most famous Baijiu) and Huandiaonuo (used for Fen-flavor Baijiu) (Bao et al., 2024). T2T resources provide insights into dark genomic regions (e.g., centromeres/telomeres), the highest-resolution genetic map for Baijiu-trait breeding, and a model for T2T assemblies in other plants. Comparative genomic analysis reconstructed a 65-gene metabolic pathway for tannin synthesis (including eight transcription factors, three transporters, and 45 structural genes), providing a genetic reference for Baijiu-oriented breeding (Ding et al., 2024). Three regulatory genes (*Yellow seed 1*, *TANNIN 1*, AND *TANNIN 2*) involved in tannin biosynthesis have been identified (Zhang et al., 2024b, Zhang et al., 2023b).

Recently, Chen et al. (2025) assembled a gapless, T2T reference genome (729.5 Mb) for sorghum variety E048. Comparative genomic analysis identified a 2.9 Mb E048-specific region with 187 genes related to signal transduction, immune response, and metabolic regulation (Chen et al., 2025). This offers insights into superior agronomic traits like disease resistance, stress tolerance, lodging resistance, and high sugar content. They developed an EMS mutant library comprising 13,226 M_1_ plants (covering 97.54% of genes), established efficient MutMap/MapMap+ gene mapping methods, and optimized an *Agrobacterium*-mediated transformation system. These resources provide a “genome-mutant-transformation” pipeline for functional genomics and molecular breeding in sorghum. All data have been integrated into the Sorghum Genome and Mutant Database (SGMD; https://sorghum.genetics.ac.cn/SGMD), creating a comprehensive research platform that links genome analysis and breeding applications (**Table 1**) (Chen et al., 2025).

### 5.3 Pan-genome unlocks sorghum genetic diversity and breeding innovations

A single reference genome cannot capture the full genetic diversity of a species, limiting exploration of genetic variations. Additionally, restricted diversity from recombination in elite breeding populations may fail to address environmental challenges. The pangenome of a species consists of all its genes and is essential for understanding variation within it. It has three components: (1) core genome (genes shared by all individuals), (2) dispensable genome (genes present in some individuals), and (3) strain-specific genes (unique to single strains) (Tao et al., 2021a). A representative sorghum pan-genome had been published in 2021 (Tao et al., 2021a). Tao et al. (2021a) constructed the first broadly representative sorghum pan-genome using 13 varieties, including *S. propinquum*, wild sorghum, and cultivated sorghum. They employed multiple omics technologies, including second-generation sequencing, third-generation sequencing, Hi-C, and transcriptomics, achieving a maximum contig N50 of 3.48 Mb for genome assembly. This sorghum pan-genome size is 954.8 Mb, 30% larger than the reference genome (BTx623, 732.2 Mb), with core sequences accounting for 62% and extensive presence-absence variations (PAVs) across genomes. Combining pan-genome data, they performed GWAS on grain color and identified a 3,216 bp PAV in the Yellow seed1 gene. *SbRC*, homologous to the rice grain color gene *Rc*, showed a 416 bp PAV in the pan-genome.

Ruperao et al. (2021) developed a more comprehensive sorghum pan-genome using reference genomes and 354 genetically diverse sorghum accessions representing different races. They identified 35,719 genes, with a core genome of 16,821 and an average of 32,795 per cultivar. Notably, 53% of genes exhibited presence-absence variation, with variable genes enriched for environmental responsiveness and capable of classifying accessions by race. Association analysis using over two million SNPs from the pan-genome identified 398 significant SNPs linked to agronomic traits. The expression analysis of drought-responsive genes revealed 1,788 are functionally essential, including 79 absent from the BTx623 genome, providing valuable genomic resources linking genetic diversity to adaptive traits, particularly drought response (Ruperao et al., 2021). Marla et al. (2025) recently analyzed a pan-genome derived from 1,661 sorghum germplasm resources and identified seven loss-of-function variants in the *DW3* gene. A 137 bp deletion *Dw3* allele from the variety Segaolane showed excellent properties for suppressing plant height reversion mutations (Marla et al., 2025). This study provides an innovative solution to the long-standing problem of height reversion mutations in U.S. grain sorghum breeding. The sorghum pan-genome will revolutionize research by integrating GWAS of key traits with structural variation analysis, advancing our understanding of sorghum functional genomics and breeding (**Table 1**) (Poosapati et al., 2022).

### 5.4 Multi-omics and the database platform for functional genomics

Multi-omics involves studying organisms at multiple molecular levels, such as genomics, transcriptomics, proteomics, and metabolomics. SorghumFDB (http://structuralbiology.cau.edu.cn/sorghum/) is a functional genomics data mining platform that provides comprehensive functional annotations of genes (Tian et al., 2016). Liu et al (2021b) integrated whole-genome SNP and INDEL variation data from 289 sorghum accessions based on the BTx623 (v3.1) reference genome, establishing the multi-omics platform SorGSD (https://ngdc.cncb.ac.cn/sorgsd/). It integrates phenotypic traits and panicle morphology images for genotype-phenotype co-analysis. It uses tools like ID conversion, gene alignment, and genome browsers, enabling efficient multi-omics data mining for sorghum functional genomics and molecular breeding (Liu et al., 2021b). Bao et al. (2024) established the first brewing sorghum genome database (http://sorghum.org.cn/) featuring genome browsing, sequence alignment, chromosomal collinearity analysis, and data download (**Table 1**) (Bao et al., 2024). Chen et al. (2025) developed the Sorghum Genomics and Mutant Database (SGMD, https://sorghum.genetics.ac.cn/SGMD) by integrating genomic data, a gene expression atlas, and mutant variations, serving as a “super toolkit” for sorghum functional gene research (**Table 1**) (Chen et al., 2025).

Breakthroughs have been made in understanding sorghum stress resistance mechanisms through multi-omics approaches. Jiao et al. (2023) used multi-omics analysis to identify 2,683 differentially expressed genes and 160 metabolites, offering insights into crop cadmium resistance mechanisms (Jiao et al., 2023). Li et al. (2024a) revealed that drought and salt stress responses involved whole-genome duplication and conserved domains in sorghum transcription factors, while transcriptomics identified 45 key genes. RNA and degradome analyses revealed miR5072 and its target gene (*Sobic.001G449600*) related to drought resistance, while WGCNA further identified drought-responsive genes. Ultimately, 15 candidate genes were found, including two TFs: HD-ZIP family *Sobic.004G300300* and bZIP family *Sobic.003G244100* (Li et al., 2024c). Liu et al. (2024) integrated genomics and transcriptomics with phenotypic and physiological analyses to elucidate nitrogen use efficiency (NUE) mechanisms in sorghum. Co-expression network analysis identified key genes like nitrogen transporter *Sobic.003G371000.v3.2leaf* (*NPF5.10*) and transcription factor *Sobic.002G202800.v3.2leaf* (*WRKY*) that enhance NUE by regulating nitrogen uptake (NUpE) and utilization efficiency (NUtE) under low-nitrogen stress, presenting vital targets for developing N-efficient sorghum varieties (Liu et al., 2024). Seitz et al. (2024) used metabolomics and metatranscriptomics to analyze how root exudates from four crops (sorghum, hairy vetch, rapeseed, and rye) regulate soil microbiome functions. They created the first genomic database on crop-microbiome interactions, which helps to understand soil biogeochemical processes (Seitz et al., 2024). Mishra et al. (2021) studied micronutrient responses in sorghum under iron (Fe) and zinc (Zn) deficiency/excess by integrating transcriptomics and ionomics. RNA-seq revealed transcriptional regulation in roots and leaves during stress, with Fe deficiency and Zn excess causing notable phenotypic and gene expression changes. Gene regulatory network (GRN) analysis identified hub genes (*SbFIT*, *SbPYE*, *SbBTS*) in roots that regulate Fe/Zn uptake, while leaf homologs primarily influence chloroplast function, photosynthesis, and oxidative stress. Xing et al. (2025) used multi-omics analysis to show how spermidine (Spd) improves vigor in aged sorghum seeds via antioxidant networks. Integrated analysis of transcriptomics, proteomics, and metabolomics shows that Spd enhances antioxidant enzyme activity and metabolite accumulation, clearing ROS and reversing seed aging effects (Xing et al., 2025).

Shi et al. (2024) developed HEMU (The HEMU Andropogoneae Database), the first integrated multi-omics analysis platform for Andropogoneae grasses, incorporating nearly 5,000 multi-omics datasets across 20 species (including maize, sorghum, and sugarcane). HEMU covers 75 genomes, 4,718 RNA-seq datasets (1,527 maize, 1,428 sorghum), 90 ChIP-seq epigenomic datasets (14 tissues, 10 histone modifications), and 37 ATAC-seq datasets (13 tissues). Its innovative six-toolkit system (genomics, transcriptomics, epigenomics, gene families, transposable elements, and integrated analysis) enables multi-level, one-stop analysis from DNA sequences to epigenetic modifications, providing powerful multi-omics support for functional gene discovery and molecular breeding in Andropogoneae crops (Zhu et al., 2024). Ko and Brandizzi (2025) developed NEEDLE (https://github.com/DaeKwan-Ko/needle) to tackle analytical challenges in non-model crops. This tool integrates dynamic transcriptome data to construct co-expression network modules and identify key transcriptional regulators, successfully analyzing cellulose synthase-like F6 (*CSLF6*) in sorghum and Brachypodium (**Table 1**) (Ko and Brandizzi, 2025).

## 6 Application of genetic innovations in sorghum

Trait-specific genetic innovations are central to improving sorghum’s agronomic performance and climate resilience. Key traits, such as plant height, grain yield, protein digestibility, drought and salt tolerance, and resistance to pests and diseases, have been extensively explored in sorghum research communities. By linking each trait to its underlying gene(s) and associated discoveries, molecular breeding and functional genomics are being investigated to address challenges posed by climate change and food insecurity. These insights provide valuable targets for developing elite sorghum cultivars tailored to diverse agroecological conditions (**Table 2**).

**Table 2:** Application of genetic innovations in sorghum.

Key trait	Gene symbol	Major findings	References
Plant height regulation	*Dw1-Dw4*	Identified four dwarfing loci controlling internode length	(Quinby and Karper, 1954)
	*Dw1-Dw4*	Found complete absence of dw2 allele in key Chinese breeding materials	(Wang et al., 2024)
	*qHT7.1* (encoding a MYB transcription factor)	Transposon insertion (740bp) causes aberrant splicing, leading to dwarf phenotype	(Mu et al., 2024)
Grain size optimization	*SbDEP1*	Key gene balancing grain number per panicle and grain weight	(Tao et al., 2021b)
Grain number per panicle	*MSD1* (TCP-family transcription factor)	First evidence of JA signaling pathway regulating panicle development	(Jiao et al., 2018)
	*DG1* locus	Regulates floret development via histone modifications	(Zhang et al., 2025a)
Seed shattering reduction	SH1 (YABBY transcription factor)	Three independent domestication haplotypes; 2.2-kb deletion reduces harvest loss by 80%	(Lin et al., 2012)
Seed hull enclosure	*GC1* (Gγ subunit)	Modulates glume cell proliferation by degrading pPLAII-1 phosphatase	(Xie et al., 2022)
Optimizing plant architecture	*LG1*	Monoallelic mutants enhance leaf erectness; biallelic mutants eliminate ligules	(Brant et al., 2021)
Shade Avoidance	*phyB1/B2-LG1-HB53* Module	Conserved mechanism of phytochrome-coordinated architecture regulation	(Shi et al., 2024)
Panicle neck elongation	*shp-I* (a BTB/POZ and MATH domain protein)	BR and auxin synergistically regulate parenchyma cell size in neck internodes	(Ao et al., 2025)
Dual-pathway tillering regulation	*phyB*, *SbTB1*	Active phytochrome B (Pfr) inhibits SbTB1 to promote tillering, while inactive phyB enhances SbTB1 to suppress tillering	(Kebrom et al., 2006)
Strigolactone signaling pathway	*SbMAX2*	Defoliation regulates tillering through the SbMAX2-mediated strigolactone signaling pathway	(Kebrom et al., 2010)
Key tillering regulator	*NAB1* (encoding CCD7 enzyme)	NAB1 mutation disrupts strigolactone biosynthesis and enhances auxin transport, increasing tillering	(Chen et al., 2018)
Major QTL for tiller number	*TIN1* (a C2H2 zinc finger transcription factor)	The C2H2 zinc finger transcription factor TIN1 regulates tillering by suppressing GT1/LABA1/AN-2 expression and interacting with TOPLESS proteins	(Zhang et al., 2019)
Genome-wide association study	Multiple QTNs	Identified multiple stable quantitative trait nucleotides (QTNs) for tiller number through GWAS	(Wang et al., 2022; Wondimu et al., 2023)
Core tillering locus	*Sobic.001G152700* (encoding a DUF1618 protein)	DUF1618-domain gene is the only horizontally transferred gene from sorghum to Striga hermonthica	(Upadhyaya et al., 2024)
Panicle morphology adaptation	*Sobic.003G052700* (QTL3.4721839); *Sobic.006G247700* (QTL6.58709500)	Two domestication genes showing geographical selection patterns - long loose panicles in southern China vs compact panicles in northern regions	(Zou et al., 2024)
Energy metabolism coordination	*Sobic.006G061100* (*SbSNF4-2*)	AMPK/SNF1/SnRK1 γ-subunit overexpression increases biomass, delays flowering and enhances sugar content	(Upadhyaya et al., 2022)
Rhizome-yield relationship	20 SSR markers in 8 genomic regions	Challenges yield-rhizome trade-off paradigm; shows positive correlation (heritability 0.723) and identifies 5 novel loci	(Zheng et al., 2025)
Tannin biosynthesis regulation	*TAN1* (Sobic.004G280800)	The *tan1-e* appears exclusively in Chinese landraces, while other alleles are globally distributed	(Wu et al., 2012; Zhang et al., 2024c)
	*TAN2* (Sobic.002G076600)	The tan2-d allele was predominantly selected in China	(Wu et al., 2019; Zhang et al., 2024c)
	*Y1* (Yellow seed 1, Sobic.001G398100)	MYB transcription factor regulating pericarp pigmentation and 3-deoxyanthocyanidin accumulation	(Tao et al., 2021a)
	73 QTLs	Colocalization with flavonoid biosynthesis genes homologous to Arabidopsis (TT2, TT7 etc.) and rice (MYB61)	(Ni et al., 2025; Zhang et al., 2023b)
Protein digestibility improvement	*k1C* gene family	CRISPR/Cas9 editing of α-kafirins improved protein digestibility and lysine content	(Li et al., 2018)
	Synthetic β-kafirin gene	Engineered protease cleavage sites increased protein content (11-37%) and digestibility (11-21%)	(Liu et al., 2019)
	*K2G* (γ-kafirin)	Mutations in signal peptide reduced γ-kafirin 12.75-19.22%, improved digestibility 26.91-74.31%	(Li et al., 2023)
Improving water use efficiency	*SbPIP1.1*/*SbTIP3.2*	Aquaporin haplotypes significantly associated with intrinsic water use efficiency (iWUE)	(Al-Salman et al., 2024)
	*SbEPFsyn* (based on *EPF2* and *EPF9*)	Synthetic biology-designed stomatal patterning gene reduced stomatal density, increasing soil water retention by 50%	(Ferguson et al., 2024)
Drought resistance	*BM41* (a transmembrane protein kinase)	Encodes a leucine-rich transmembrane protein kinase that positively regulates VLCFA synthesis; bm41 mutant shows significantly reduced wax content	(Tian et al., 2024)
	*Stg1-Stg4* QTL	Four QTLs collectively explain 54% of phenotypic variation	(Borrell et al., 2014)
	*SbPIN2*/*SbPIN4*	Auxin efflux carriers regulating root architecture and panicle development, significantly increasing yield under drought	(Borrell et al., 2022)
	*SbWRKY30*	Activates stress-responsive genes by binding to W-box in SbRD19 promoter	(Yang et al., 2020)
	*SbNAC014/034/035/037/041*; *SbNAC052/073/116*	SbNAC014/034/035/037/041, positively regulate post-flowering drought responses, while SbNAC052/073/116 are negative	(Sanjari et al., 2019)
	*SbGH3*, *SbLBD*	Auxin response factors (ARFs) participate in drought response by regulating target genes such as SbGH3 and SbLBD	(Wang et al., 2010)
	*SbAO3*, *SbCIPK15*, *SbMAPK10*	ABA enhances drought tolerance by inducing stomatal closure and activating stress-responsive genes	(Abou-Elwafa and Shehzad, 2018; Wang et al., 2016)
	*SbBRI1/SbBES1*	SbBRI1: Blocks drought responses; mutations increase tolerance; SbBES1: Normally makes lignin, switches to flavonoids in drought	(Fontanet-Manzaneque et al., 2024a)
	*SbCKX4*	Overexpression increases root biomass and drought resistance	(Pospíšilová et al., 2016)
	*SbER2-1*	Leucine-rich repeat receptor-like kinase maintains photosynthetic rates and delays leaf senescence	(Li et al., 2019)
Salt-alkali tolerance mechanisms	*SORBI_3004G304700*	L-type lectin receptor-like kinase family negative regulator; haplotype 1 potentially dominant for salt adaptation	(Mao et al., 2024)
	*SbWRKY50*	SbWRKY50 negatively regulates salt response by altering ion homeostasis	(Song et al., 2020)
	*SbWRKY55*	Reduces ABA levels by suppressing SbBGLU22, interacts with SbFYVE1 to block ABA signaling	(Song et al., 2022)
	*AT1* (homologous to rice GS3)	Atypical G-protein γ subunit regulating H_2_O_2_ efflux; loss-of-function mutations increase yield by 20-30% in saline-alkali soils	(Zhang et al., 2023a)
Disease resistance	*ARG1* (NLR protein)	MITE-transposon regulated NLR confers complete broad-spectrum resistance; truncated ARG1 leads to susceptibility	(Lee et al., 2022)
	*ARG2*	Activates anthocyanin/zeatin pathways without growth penalty, maintains stable resistance across temperatures	(Mewa et al., 2022)
	*ARG4/ARG5* (NLR receptors)	SAP135 and P9830 lines carry functional ARG4/ARG5 respectively; ARG4 or ARG5 and their recombinant inbred lines demonstrated resistance to strains Csgl1 and Csgrg	(Habte et al., 2024)
	*Y1/Y3* (MYB TFs)	*Y1/Y3* showing enhanced expression in resistant lines	(Nida et al., 2019)
Pest resistance	*Bmr12* loss-of-function mutant; *Bmr12*-OE	Loss-of-function mutations confer aphid resistance via IAA-Asp reprogramming; overexpression resists fall armyworm via JA pathway and flavonoid accumulation	(Grover et al., 2024; Kundu et al., 2025)
	*SgR1* (LRR-RLP protein)	Phloem-specific expression, promoter -965bp deletion and 592 SNP strongly associated with resistance	(Zhang et al., 2024a)
	*RMES1A/B* (atypical NLRs)	Activates conserved immune network (not cyanogenic glycosides), originating from ancient Poaceae gene cluster through rapid selection	(VanGessel et al., 2025)

### 6.1 Grain yield enhancement

Recent innovations in molecular biology and genomics have greatly improved our understanding of the genetic regulatory networks governing yield-related traits in sorghum. Plant height, which influences lodging resistance and biomass, has garnered widespread attention in breeding. Quinby and Karper identified four loci (*Dw1*-*Dw4*) that regulate plant height by modifying internode length (Karper and Quinby, 1947). However, the unclear allelic composition of Dw1-Dw4 genes in primary breeding materials has increased plant height in the identical height type progeny, leading to suboptimal morphological traits. Wang et al. (2024) revealed that China’s predominant sterile lines mainly exhibit the “triple-dwarf” type (*Dw1*-*Dw2*-*dw3*-*dw4*) from Kafir and its improved lines, while restorer lines are primarily composed of the improved “double-dwarf” type (*Dw1*-*Dw2*-*dw3*-*dw4*) from the Kaoliang/Caudatum subspecies, along with some Kafir-derived “triple-dwarf” types. Notably, the *dw3* allele was predominant in the tested materials, whereas *dw1* occurred less frequently in the restorer lines. Importantly, the dw2 allele, which significantly influences plant architecture, was completely absent in key restorer materials. These findings highlight the need for precise genotyping of Dw1 and Dw2 alleles to enable differentiated breeding strategies, offering theoretical and technical support for marker-assisted dwarf breeding sorghum (**Table 2**) (Wang et al., 2024). Complementing the *Dw1*-*Dw4* system, several other genes regulating plant height have been identified in sorghum, which controlplant height by regulating internode elongation and cell proliferation. A 740-bp transposable element insertion in *qHT7.1* (encoding a MYB transcription factor) intron leads to aberrant splicing and premature termination, resulting in a dwarf phenotype in sorghum.

Grain size variation is a major determinant of yield and quality in cereal crops. It is governed by both the plant’s genetic potential and the availability of assimilates allocated for grain filling. Tao et al. (2021a) found that five grain-size-related parameters exhibited high heritability, and artificially reducing grain number led to increased grain weight. The GWAS analysis identified 94 QTLs, with SbDEP1 confirmed to balance grain number per panicle and grain weight by regulating primary branch number. It provided insights on “source-sink” relationships, showing that grain size is influenced by genetic potential and assimilate partitioning, highlighting key targets for improving yield components in cereals (**Table 2**) (Tao et al., 2021b).

Grain number per panicle is another critical yield determinant. Jiao et al. (2018) obtained a multi-seeded sorghum mutant (*msd1*) through EMS mutagenesis and discovered that *MSD1*, a TCP-family transcription factor, influences panicle development via the jasmonic acid (JA) pathway. The mutant exhibited a 50% reduction in JA content in young panicles, and exogenous JA application restored the phenotype. This finding establishes the first link between JA signaling and panicle architecture (Jiao et al., 2018). The DG1 locus promotes lower floret development by modulating histone modifications, leading to a double-grain trait. These studies elucidate molecular mechanisms of inflorescence development and highlight the unique value of multi-grain sorghum in brewing (**Table 2**) (Zhang et al., 2025a).

Seed shattering is a trait that directly impacts harvesting efficiency. Lin et al. (2012) discovered that seed shattering is controlled by a single gene, *SHATTERING1* (*SH1*), which encodes a YABBY transcription factor in sorghum. Domesticated sorghum carries three distinct mutations at the *SH1* locus, and variations in the promoter and intronic regulatory regions result in low expression levels. A 2.2-kb deletion leads to a truncated transcript lacking exons 2 and 3. A GT-to-GG splice-site mutation in intron 4 causes the exclusion of exon 4. *SH1* underwent parallel selection during the domestication of sorghum, rice, and maize. Notably, the 2.2-kb deletion mutation in 80% of cultivated varieties significantly reduces harvest losses, providing molecular evidence for understanding crop domestication (**Table 2**) (Lin et al., 2012).

Threshing efficiency in Poaceae crops relates closely to seed hull enclosure. Cereal crops like sorghum, rice, and wheat typically have seeds enclosed by glumes, with hull loss marking a significant event in panicle domestication. Sorghum exhibits rich phenotypic variation in hull enclosure. GWAS analysis identified GC1 as a negative regulator of hull enclosure, encoding an atypical G*γ* subunit. GC1 modulates hull cell proliferation; overexpression reduces hull enclosure, while knockout enhances it. GC1 interacts with phosphatase pPLAII-1, promoting its degradation to regulate glume development. *gc1* allelic variants are present in 40% of sorghum germplasms, indicating strong artificial selection for this beneficial characteristic (**Table 2**) (Xie et al., 2022).

Optimizing plant architecture is a critical pathway for achieving yield gains. Studies in sorghum (*Sorghum bicolor* L. Moench) show that high-density planting of erect hybrid varieties mainly drives increased yields. Through CRISPR/Cas9, researchers successfully generated monoallelic and biallelic mutants of the *LG1* gene in sorghum. Monoallelic mutants exhibited enhanced leaf erectness, while biallelic mutants lacked ligule structures and showed further reduced leaf angles (Brant et al., 2021). However, high-density planting triggers shade avoidance responses (SAR) that optimize light capture but compromise plant vigor and ultimately limit yield potential. The mechanism behind this phenomenon is that phyB1/B2 serves as the primary photoreceptor, detecting changes in the ratio of red (R) to far-red (FR) light and coordinating plant responses through the LG1-HB53 regulatory module (Shi et al., 2024). Plant architecture influences light-use efficiency in sorghum and closely correlates with panicle development. Panicle neck elongation is a critical yield factor. The *sheathed panicle-I* (*shp-I*) mutant showed shortened neck internodes due to reduced parenchyma cell size. A single recessive gene, *SbiHYZ.10G230700*, controls this trait, encoding a BTB/POZ and MATH domain protein. Intriguingly, the mutant also exhibited reduced auxin levels and elevated brassinosteroids, suggesting synergistic hormonal regulation of panicle neck development (**Table 2**) (Ao et al., 2025).

Tillering directly affects plant structure and yield formation. A dual-pathway regulatory model was established for *Sorghum* tillering (Kebrom et al., 2010, Kebrom et al., 2006). Kebrom et al. (2006) first revealed the phyB-SbTB1 module that controls sorghum tillering, where the active form of phytochrome B (*phyB*, Pfr) inhibits *SbTB1* to promote tiller bud, while inactive phyB increases *SbTB1* to suppress tillering (Kebrom et al., 2006). Later, a separate pathway was identified where defoliation regulates tillering through the *SbMAX2* gene (an *Arabidopsis MAX2* homolog) mediated strigolactone signaling pathway (Kebrom et al., 2010). Chen et al. (2018) added *NAB1* (encoding CCD7 enzyme) as a crucial regulator; its mutant increases tillering due to disrupted strigolactone biosynthesis and enhanced auxin polar transport (Zhang et al., 2019). Regarding transcriptional regulation, comparative analysis revealed the conserved function in grasses of the *TIN1* gene, which encodes a C2H2 zinc finger transcription factor to regulate tillering by suppressing *gt1* and Laba1/An-2 expression and interacting with TOPLESS proteins. A major QTL for tillering number was identified in the *TIN1* region in sorghum (Zhang et al., 2019). Subsequent QTL mapping identified regions with differentially expressed genes (DEGs), including potential regulators like *DRM1* and *WUSCHEL*. With advances in high-throughput sequencing technologies, researchers have begun to unravel the genetic basis of tillering traits at the whole-genome level. GWAS (Wang et al., 2022; Wondimu et al., 2023) using SNP markers identified multiple stable QTNs for tiller number (Wang et al., 2022; Wondimu et al., 2023). Upadhyaya et al. (2024) evaluated a mini-core sorghum collection and found a consistently detected tillering (TL) locus on chromosome 1, containing *Sobic.001G152700* (encoding a DUF1618 protein). This gene is the only gene horizontally transferred from *Sorghum* to the parasitic weed *Striga hermonthica*, potentially related to environmental adaptation (Upadhyaya et al., 2024).

Panicle morphology in sorghum influences its grain yield and resistance to pests and diseases. Panicle morphology traits include length, rachis node number, primary branch number, maximum primary branch length, and compactness. GWAS identified that 71 QTLs were distributed across 41 genomic regions on 9 chromosomes using a sorghum population adapted to diverse environments in China (Zou et al., 2024). Two domestication-related genes (*Sobic.003G052700* and *Sobic.006G247700*) were located within two major QTL regions (*QTL3.4721839* and *QTL6.58709500*) detected across multiple environments. The allelic variations of these genes exhibited a geographical pattern, suggesting that sorghum breeders in southern and northern China have selected for different panicle morphology traits. Southern sorghum varieties have long, loose panicles, adapting to hot, humid climates, while northern varieties exhibit short, compact panicles, enabling higher planting density and greater grain yield in arid regions (Zou et al., 2024). This work offers new breeding strategies and resources for developing sorghum suited to local conditions.

Plant height and branching characteristics jointly determine canopy structure and light-use efficiency. The genetic regulation of energy metabolism pathways can coordinate these architectural traits with reproductive growth. Upadhyaya et al. (2022) conducted a GWAS analysis using a sorghum mini-core collection and identified multiple QTLs for days to flowering, plant height, biomass, and sugar content. Notably, overexpression of *Sobic.006G061100* (*SbSNF4-2*, encoding the *γ*-subunit of the AMPK/SNF1/SnRK1 complex) in both sorghum and sugarcane significantly increased biomass and plant height while delaying flowering and enhancing sugar content. It was revealed how energy-sensing pathways integrate plant development with carbon partitioning, providing key targets for achieving an ideal plant type characterized by “high biomass-high sugar-moderately late flowering” (Upadhyaya et al., 2022).

Importantly, yield improvement relies not only on aboveground architectural optimization but also on belowground organ functionality. Conventional studies suggest a trade-off between rhizomes (as carbon storage organs in perennial crops) and grain yield. However, Zheng et al. found that rhizome biomass exhibits high heritability (0.723) and strong positive correlations with total belowground biomass (r1 = 0.95; r2 = 0.97). A positive correlation was found between rhizome biomass and grain yield, potentially mediated by rhizome-enhanced tillering effects. Through bulked segregant analysis (BSA), researchers mapped 20 SSR markers linked to rhizome traits in 8 genomic regions, including 5 novel loci, and selected elite lines with high rhizome biomass, biomass yield, and grain yield (Zheng et al., 2025). This discovery challenges traditional paradigms, proving that we can improve sorghum’s carbon sequestration and agronomic yield through genetic breeding.

### 6.2 Nutritional quality improvement

The nutritional value of sorghum is limited by three factors: lack of essential amino acids (notably lysine), low protein digestibility, and insufficient sugar and oil in traditional varieties (Duodu et al., 2003). Nutritional shortcomings have caused widespread “hidden hunger” in areas where sorghum is a staple—malnutrition marked by adequate energy intake but micronutrient deficiencies. Enhancing sorghum’s nutrition via molecular breeding and metabolic engineering has become a new frontier in global agricultural research.

#### 6.2.1 Low-tannin sorghum

Plants synthesize flavonoids like flavonols, anthocyanins, and proanthocyanidins, also known as condensed tannins. Sorghum’s high condensed tannin content affects seed dormancy, grain mold resistance, and protection against bird and insect predation. Moreover, it significantly influences the taste and flavor of Chinese distilled liquors (Marla et al., 2025) Three key regulatory genes have been identified in sorghum: *TAN1* (*Sobic.004G280800*), encoding a WD40 protein corresponding to the *B2* locus and homologous to Arabidopsis *TTG1* (Wu et al., 2012); *TAN2* (*Sobic.002G076600*), containing a bHLH domain corresponding to the B1 locus and homologous to Arabidopsis *TT8*, rice *Rc*, and maize *IN1* (Wu et al., 2019); and the MYB transcription factor *Y1* (*Sobic.001G398100*), which regulates pericarp pigmentation and 3-deoxyanthocyanidin accumulation (Tao et al., 2021b). *Tan1* and *Tan2* are conserved regulators in the tannin biosynthesis pathway, exhibiting high nucleotide similarity in major cereal crops that produce grain tannins. Seven and eight recessive alleles controlling tannin absence have been identified in the *Tannin1* and *Tannin2* genes, respectively, across sorghum varieties.

Recently, Zhang et al (2024b) identified two novel recessive alleles from 421 sorghum accessions: *tan1-d* with a 12-bp deletion at 659 nt and *tan1-e* with a 10-bp deletion (CGACATACGT) between positions 771-780 (Zhang et al., 2024c). The *tan1-d* allele shows sequence variations similar to *tan1-c* (A-to-T inversion at 1054 nt, GT deletion at 1057–1058 nt, and C-to-T transition at 1059 nt) that cause frameshift mutation of the TGA stop codon (positions 1060-1062), resulting in a nonfunctional protein despite retaining four WD-40 domains. The *tan1-e* 10-bp deletion induces a frameshift, producing a truncated 295-aa protein with an altered fourth WD-40 domain. *Tan1-e* occurs only in Chinese landraces, whereas other alleles like *tan1-a* and *tan1-b* are globally distributed and absent in Chinese landraces (Zhang et al., 2024b). Zhang et al. (2024) discovered *tan2-d* allele with a C-to-T transition creating a premature stop codon before the bHLH domain, showing strong selection in Chinese germplasm (Zhang et al., 2024c). They also identified four novel alleles, with three alleles (*tan2-d*, *tan2-e*, *tan2-f*) disrupting the bHLH domain and losing functionality. In contrast, *tan2-g*, a null allele, exhibits nucleotide substitutions and insertions between positions 1579–1607 in the coding region but unexpectedly retains an intact bHLH domain. Among these, *tan2-e* is uniquely present in Chinese landraces (**Table 2**) (Zhang et al., 2024b).

Tannin content is also associated with grain color (e.g., reddish-brown grains typically have higher tannins). 73 QTL were detected to be associated with grain pericarp (exocarp/mesocarp) color, testa pigmentation, and tannin content (47 potentially novel), with key QTL colocalizing with flavonoid biosynthesis pathway genes homologous to *Arabidopsis* (*TT2*, *TT7*, *TT12*, *TT16*, and *AT5G41220/GST*) and rice (*MYB61*, *OsbHLH025*) (Zhang et al., 2023b). Ni et al. (2025) used metabolomics to identify metabolites and pathways explaining quality differences between “Hongyingzi” (HYZ) sorghum and four varieties (“Jinuoliang”, “Jinnuoliang”, “Lunuohong”, “Liaoza 19”), emphasizing ellagic acid-4-O-glucoside’s role in tannin synthesis and offering insights into key genes in flavonoid metabolism in sorghum seeds (Ni et al., 2025).

#### 6.2.2 Palatability and digestibility improvement

Sorghum grain has 10-12% protein, but imbalanced essential amino acids and low digestibility limit its nutritional value. The resistant *γ*- and *β*-kafirins form outer layers that encapsulate *α*-kafirins, leading to poor digestibility of sorghum grains. A GWAS analysis reveals natural variation in sorghum protein content, with Ethiopian and Indian Durra types showing the highest levels (8.1-18.8%) (Rhodes et al., 2017). Due to their unique structure, Kafirins, comprising 70-80% of grain protein, have low digestibility. Researchers have developed two strategies: Li et al. (2018) used CRISPR/Cas9 to target the α-kafirin-encoding *k1C* gene family, significantly improving protein digestibility and lysine content in T_2_ generation lines (Li et al., 2018); They also employed synthetic biology to design a *β*-kafirin gene with 10 additional protease cleavage sites, resulting in transgenic lines with 11-37% higher protein content and 11-21% improved pepsin digestibility (**Table 2**) (Liu et al., 2019). Li et al. (2023) designed sgRNA targeting the *K2G* gene (encoding *γ*-kafirin) on chromosome 2, introducing mutations in the endoplasmic reticulum signal peptide coding region, leading to a 12.75%-19.22% reduction in *γ*-kafirin content and a 26.91%-74.31% improvement in raw flour protein digestibility in the mutant seeds (Li et al., 2023). Broiler feeding trials demonstrated that high-protein transgenic sorghum (154.7 g/kg) reduced soybean meal usage by 20.5% and increased breast meat yield by 6.33%, confirming its nutritional benefits (**Table 2**) (Macelline et al., 2024).

### 6.3 The abiotic stress and climate change adaptation

#### 6.3.1 Water-efficient sorghum

Sorghum is drought-resistant, but its production is vulnerable to water scarcity during critical growth stages like flowering and grain filling (Xu et al., 2000). Recent research revealed that haplotypes of aquaporin genes *SbPIP1.1* and *SbTIP3.2* are significantly associated with intrinsic water-use efficiency (iWUE) in sorghum, independent of leaf hydraulic conductance (Al-Salman et al., 2024). Ferguson et al. (2024) pioneered a synthetic biology approach by expressing an artificially designed epidermal patterning factor gene (*SbEPFsyn*, based on *EPF2* and *EPF9*), developing new sorghum lines with significantly reduced stomatal density. The transgenic plants displayed higher iWUE and better drought performance: soil water retention rose by 50%, enhancing photosynthetic activity and cell turgor (Ferguson et al., 2024). Reduced stomatal density resulted in abnormal panicle development and yield loss, emphasizing the need for future research to balance water efficiency and reproductive growth. These findings identify molecular targets for drought-resistant breeding in C_4_ crops, emphasizing genetic improvements for better water efficiency.

#### 6.3.2 Drought resistance

Under drought and heat stress conditions, leaves increase heat shock proteins (HSPs) and antioxidants, while roots activate osmoprotectants and repair pathways (Xu et al., 2000). Tolerant sorghum genotypes under drought, salinity, and alkalinity stress show increased antioxidants, including catalase and superoxide dismutase (SOD), and osmoprotectants like proline and glycine betaine. In contrast, sensitive varieties exhibit severe effects like membrane damage and chlorophyll degradation. The cuticular wax (CW) serves as the primary barrier against drought. The sorghum *bm41* mutant exhibits a significantly reduced cuticular wax content and very-long-chain fatty acids (VLCFAs). The *BM41* gene encodes a transmembrane protein kinase rich in leucine, acting as a positive regulator of the wax biosynthesis gene *KCS6* (Tian et al., 2024). The discovery offers a promising target for breeding drought-tolerant varieties.

The stay-green trait is crucial for sorghum to manage terminal drought stress (Ochieng et al., 2020). This trait, characterized by delayed leaf senescence, prolongs photosynthetic activity, reduces lodging risk, and enhances grain yield. Four QTLs (*Stg1*, *Stg2*, *Stg3*, and *Stg4*) have been identified in sorghum for the “stay green” phenotype, collectively accounting for nearly 54% of the phenotypic variance (Borrell et al., 2014). Borrell et al. (2022) demonstrated that the auxin efflux carrier genes *SbPIN4* (located in *STG1*) and *SbPIN2* (located in *STG2*) are key regulators of the stay-green phenotype, and transgenic studies showed these genes influence canopy architecture, root development, and panicle growth, enhancing yield under drought conditions. Crop simulation models predict significant yield benefits from the *SbPIN2* phenotype in arid environments (**Table 2**) (Borrell et al., 2022).

Transcription factor families like *NAC*, *WRKY*, *DOF*, and *ARF*,and *SbWRKY30*, are key regulators of drought resistance in sorghum, activates downstream stress-responsive genes by binding to the W-box in the *SbRD19* promoter (Yang et al., 2020). Other members, such as *SbWRKY45*, *SbWRKY79*, *SbWRKY83*, and *SbWRKY16*, are significantly upregulated under drought conditions, collectively forming a drought-responsive regulatory network (Baillo et al., 2020). NAC family members, *SbNAC014/034/035/037/041*, positively regulate post-flowering drought responses, while *SbNAC052/073/116* are negative (Sanjari et al., 2019). Their expression patterns vary: *NAC*, *HSF*, and *ERF* are upregulated in leaves, while expression in roots is low under mild to moderate drought stress, likely due to roots prioritizing essential physiological functions (Baillo et al., 2019). Furthermore, *SbDOF12/19/24* are rapidly activated during early drought stress, whereas S*bDOF21-23/25/27/28* respond only at later stages (Gupta et al., 2016).

Plant hormones are central to sorghum’s drought resistance, particularly auxin and abscisic acid (ABA) networks. Drought stress significantly alters the expression patterns of auxin efflux carrier genes SbPINs: *SbPIN5/8/9/11* are induced while *SbPIN3/6/7/10* are suppressed (Wang et al., 2010). These changes optimize root architecture by modulating auxin distribution, promoting deeper water uptake. Additionally, auxin response factors (ARFs) participate in drought response by regulating target genes such as *SbGH3* and *SbLBD* (Gupta et al., 2016; Wang et al., 2010). ABA enhances drought tolerance by inducing stomatal closure and activating stress-responsive genes (e.g., *SbAO3*, *SbCIPK15*, *SbMAPK10*) (Abou-Elwafa and Shehzad, 2018; Wang et al., 2016). *SbBRI1* (a brassinosteroid receptor) and its downstream transcription factor *SbBES1* in the ABA signaling pathway exhibit dual functions: under normal conditions, they promote lignin synthesis for structural stability; during drought, reduced BES1 activity activates flavonoid biosynthesis, enhancing photoprotection and photosynthetic efficiency. (Fontanet-Manzaneque et al., 2024b). Furthermore, overexpression of cytokinin (CK) metabolic enzyme gene *SbCKX4* can increase root biomass and drought resistance (Pospíšilová et al., 2016), while *SbER2-1* (a leucine-rich repeat receptor-like kinase) demonstrates higher photosynthetic rates and lignin content, delaying drought-induced leaf senescence (Li et al., 2019). These findings indicate that precisely regulating hormone pathways is essential for drought-resistant breeding.

#### 6.3.3 Salt-alkali tolerant sorghum

As per FAO reports, an estimated 23% of cultivated land, about 3.5 × 10^8^ hectares, is affected by salinity, with global saline-alkali soils covering 412 Mha (Abbas et al., 2013). The effects of salt stress include oxidative stress, hyperosmotic stress, nutrient deficiency, ion toxicity, and water loss. Plants respond to salt stress through gene expression regulation and antioxidant defence systems (e.g., ROS scavenging). Plants growing in saline-alkali regions accumulate various ionic compounds and dissolved salts.

A total of 49 *SbLLRLK* genes (L-type lectin receptor-like kinase family) were identified in sorghum, exhibiting differential expression under salt, drought, and heat stress. *SORBI_3004G304700* is a negative regulator of salt stress tolerance, with haplotype 1 potentially dominating salt adaptation. This provides a gene target and haplotype markers for molecular breeding of salt-tolerant sorghum (Mao et al., 2024).

Some genes contribute to stress tolerance in sweet sorghum. *SbWRKY50* was downregulated in M-81E (salt-tolerant) but not induced by salt stress in Roma (salt-sensitive). Overexpression of *SbWRKY50* in *Arabidopsis* demonstrated that it negatively regulates salt response by altering ion homeostasis through binding to the promoters of *SOS1* and *HKT1* (Song et al., 2020). Song et al. identified differential expression of *SbWRKY55* in M-81E and Roma under salt stress. SbWRKY55 transcriptionally represses *SbBGLU22*, reducing endogenous ABA levels. Additionally, SbWRKY55 interacts with the FYVE-type zinc finger protein (SbFYVE1), which blocks the ABA signaling pathway. SbWRKY55 is a novel component of sorghum’s salt tolerance network, opening new avenues for breeding salt-tolerant crops (**Table 2**) (Song et al., 2022).

Some sorghum varieties can even survive in soda soils with a pH as high as 10.0. Zhang et al. conducted a GWAS on 352 representative sorghum accessions and identified a major locus, *AT1*, significantly associated with alkali tolerance. *AT1* encodes an atypical G-protein *γ* subunit (homologous to rice *GS3*) that modulates plant sensitivity to alkali stress by regulating hydrogen peroxide (H_2_O_2_) efflux. Loss-of-function mutations in *AT1*/*GS3* could increase yield by 20–30% in gramineous crops grown on saline-alkali soils (pH 9.1–9.4), offering a target for improving saline-alkali land (Zhang et al., 2023a). Moreover, NAC transcription factors from wild soybean (*Glycine soja*) provide another promising strategy for enhancing stress tolerance in sweet sorghum (Zheng et al., 2023). Overexpression of the *GsNAC2* significantly enhances sorghum’s tolerance to salt-alkali (NaHCO_3_: Na_2_CO_3_ = 5:1, pH 9.63), improves shoot length, fresh weight, and water retention. GsNAC2 regulates alkali-salt tolerance by promoting positive regulators (e.g., *SbSAPK9*, *SbJAR1*) in ABA, GA, and JA pathways, while suppressing negative regulators (e.g., *SbPP2C15*, *SbRGL1*) (Wu et al., 2023). It also upregulates genes like *GCL*, *GS*, *GSH-Px*, and *GR*, boosting GSH content and antioxidant activity, mitigating oxidative damage.

### 6.4 The biotic stress and promotion of sustainable sorghum agriculture

#### 6.4.1 Disease resistance

Diseases like anthracnose (*Colletotrichum sublineolum*) and rust (*Puccinia purpurea*) significantly hinder yield—anthracnose can reduce grain yield by over 50%, whereas rust threatens late-sown crops with up to 65% yield loss (Govintharaj et al., 2025; White et al., 2012; Xu et al., 2023). Anthracnose is a major disease affecting sorghum worldwide (Schnippenkoetter et al., 2017). Highly resistant accessions can serve as valuable sources of resistance genes for breeding. Lee et al. (2022) screened natural variants of sorghum and discovered that the genotype SC283 exhibits broad-spectrum resistance to multiple strains of anthracnose fungi, while TAM428 shows susceptibility. They identified a major disease resistance locus, *ARG1* (*ANTHRACNOSE RESISTANCE GENE 1*), which encodes a canonical NLR (Nucleotide-Binding Leucine-Rich Repeat) protein. The expression of *ARG1* is regulated by a MITE (Miniature Inverted-Repeat Transposable Element) and confers complete and broad-spectrum fungal resistance. In contrast, truncated *ARG1* transcripts encoding defective NLR proteins, accompanied by elevated *NAT* expression (Lee et al., 2022). *ARG2* confers race-specific resistance to anthracnose by inducing defense responses and upregulating anthocyanin and zeatin pathways without growth trade-offs. *ARG*2 provides stable resistance across temperatures and is a promising candidate for resistance breeding. Its discovery enhances understanding of NLR-mediated defense mechanisms in sorghum (Mewa et al., 2022). Habte et al. (2024) identified the dominant resistance genes *ARG4* and *ARG5* in sorghum lines SAP135 and P9830, conferring broad-spectrum resistance against anthracnose. *ARG4* and *ARG5* encode a canonical NLR receptor. Interestingly, the sorghum lines SAP135 and P9830 each carry one functional *ARG* gene and have recessive alleles at the second locus. The resistant P9830 line contains two copies of the *ARG5* gene, while five non-functional copies exist in susceptible lines. Both resistant parental lines with either *ARG4* or *ARG5* and their recombinant inbred lines demonstrated resistance to strains Csgl1 and Csgrg, showing that these genes have overlapping specificities against different pathogen strains (**Table 2**) (Habte et al., 2024).

Enhancing crop disease resistance through genetic approaches is generally more advantageous than relying on agrochemicals. The wheat *LR34*, a single ABC transporter gene, has maintained stable resistance against rust for over a century and exhibits cross-species functionality (Krattinger et al., 2015; Risk et al., 2012). Introducing *LR34 MULTIPATHOGEN RESISTANCE GENE* (*LR34RES*) into sorghum significantly enhances resistance against two different pathogen lifestyles: transgenic lines with high *Lr34res* expression exhibit complete immunity to sorghum rust. In contrast, resistance to the hemibiotrophic sorghum anthracnose pathogen correlates with the accumulation of phytoalexins (3-deoxyanthocyanidins). LR34 activates the flavonoid biosynthesis pathway, upregulating defense-related genes within 24 hours and promoting phytoalexin accumulation, enhancing broad-spectrum resistance (Schnippenkoetter et al., 2017). Tugizimana et al. (2022) studied sorghum’s biochemical response to *Colletotrichum* infection, demonstrating that the phenylpropanoid and flavonoid pathways drive antifungal compound synthesis, such as 3-deoxyanthocyanidins, with metabolic changes dependent on time and cultivar (Tugizimana et al., 2019). Grain mold, caused by multiple pathogenic fungi, is a severe sorghum disease. A major locus with MYB transcription factors (*Y1*/*Y3*) was identified to regulate flavonoid and 3-deoxyanthocyanidin biosynthesis, showing enhanced expression in resistant lines. The *Y1* and *Y3* expression patterns in developing grains and glumes are crucial for mold resistance, though they may negatively affect traits like “injera” quality (Nida et al., 2019).

#### 6.4.2 Enhancement of pest resistance in sorghum

Sugarcane aphid (SCA) is a major pest of sorghum. Lignin plays a crucial role in plant defence against various stresses. Studies on sorghum crop pest control reveal a strong link between lignin metabolism and insect resistance. The lignin *Bmr12* loss-of-function mutant (*Bmr12*) exhibits stronger aphid resistance, whereas *Bmr12*-overexpressing (*Bmr12*-OE) lines are more susceptible (Grover et al., 2024). Aphids take longer to reach the phloem on *Bmr12* plants, indicating lignin modification influences their feeding behaviour. *Bmr12* plants experience auxin metabolic reprogramming after aphid infestation, significantly increasing indole-3-acetic acid-aspartate (IAA-Asp) levels. Exogenous application of IAA-Asp restores aphid resistance in *Bmr12*-OE plants, confirming the critical role of this metabolite in sorghum’s defence against aphids (Grover et al., 2024). Unlike aphid resistance, the *Bmr12* gene has a different regulatory mechanism for defending against the fall armyworm (FAW). *Bmr12*-OE lines show enhanced resistance to FAW, while *Bmr12* mutants are more susceptible. Interestingly, this resistance is independent of lignin content but is instead linked to FAW feeding-induced accumulation of flavonoids, which is particularly pronounced in *Bmr12*-OE plants. The jasmonic acid (JA) signaling pathway and oxidative stress response also play key roles in resistance regulation. This study reveals that *Bmr12* modulates sorghum’s defence against multiple pests through distinct metabolic pathways (Kundu et al., 2025).

The greenbug aphid is another major pest of sorghum, and cloning the resistance gene *SgR1* provides a new tool for molecular breeding (Zhang et al., 2024a). *SgR1* encodes a leucine-rich repeat receptor-like protein (LRR-RLP), with a 965 bp deletion in its promoter region and a 592 SNP in its coding sequence strongly associated with resistance. Transgenic *Arabidopsis* confirmed that *SgR1* is specifically expressed around phloem vascular bundles and is activated upon aphid infestation (greenbug biotype I (GBI)). As the first cloned GBI resistance gene, *SgR1* offers a promising target for breeding aphid-resistant sorghum varieties (Zhang et al., 2024a).

The evolutionary rescue phenomenon associated with the *RMES1* gene is particularly noteworthy. VanGessel et al. (2025) found that the sorghum aphid-resistant gene *RMES1* disrupts aphid feeding by activating a conserved plant immune network instead of via cyanogenic glycoside toxicity. This resistance is mediated by a cluster of atypical NLR immune receptor genes (RMES1A/RMES1 B). This NLR gene family originated from the evolution of an ancient gene cluster in Poaceae plants, with the resistant allele initially derived from rare natural variations in East Africa (VanGessel et al., 2025). During aphid outbreaks in the Americas, this ancient variant rapidly selected and disseminated, achieving an “evolutionary rescue” for sorghum by mobilizing the plant’s basal immune response.

## 7 Future perspectives

The future breeding for better sorghum is shaped and combined by cutting-edge biotechnologies, climate-smart strategies, digital agriculture, AI-driven breeding innovations aimed at enhancing food security in the face of climate change. CRISPR/Cas-based genome editing offers precise trait modification for yield, grain quality, disease resistance, and stress tolerance (Pacesa et al., 2024). Efforts are underway to improve editing efficiency and develop transgene-free lines to ease regulatory hurdles and increase public acceptance (Zhang et al., 2025b). Moreover, integrating genome editing with speed breeding and genomic selection can significantly accelerate breeding cycles. Developing climate-resilient sorghum varieties with stacked tolerances to drought, salinity, heat, and biotic stresses is essential, especially for smallholder farmers in Africa and Asia (Akinsemolu et al., 2024). These varieties must be tailored to local ecological systems, considering the complexity of combined stress conditions. In addition, nanobiotechnology presents novel avenues for gene delivery, stress sensing, and nutrient management (Yong et al., 2025). Nanomaterials such as carbon nanotubes and silica nanoparticles are being explored for enhancing transformation efficiency and real-time plant health monitoring. Beyond agronomic traits, breeding programs should prioritize nutritional enhancements such as improvement on protein digestibility and micronutrient accumulation (e.g., iron, zinc), and focus on expanding industrial application such as biofuels, bioplastics, and functional foods. Besides, digital agriculture, including AI-driven phenotyping and remote sensing, is revolutionizing trait analysis and crop management. Multispectral imaging platforms enable rapid, high-throughput evaluation of canopy traits such as biomass and stay-green under stress conditions. Interestingly, the creation of “ultimate sorghum phenotype” can be achieved by integrating multiple traits like deep roots, waxy leaves, and photoperiod insensitivity (Hayes et al., 2024). Recently, apomictic sorghum, also called self-reproducing hybrid or one line hybrid, has been remarkably achieved and demonstrated the potential to fix heterosis through state-of-art synthetic apomixis (Simon et al., 2025). More importantly, synthetic apomixis will dramatically reduce the hybrid cost and potentially expand the application to fix any hybrid genetic combination (Heidemann et al., 2025). Finally, policy support, infrastructure investment, and capacity building especially in developing regions, are vital for scaling up sorghum biotechnological and genetic innovations. International collaborations and public-private partnerships will be key to translating innovations into productivities in the real world.
