# Correction: Biotechnological and genetic innovations to enhance sorghum adaptation under climate change

**DOI:** 10.3389/fpls.2026.1896874

**Published:** 2026-06-16

**Authors:** Zhifang Wang, Jingzhen Wang, Ming Cheng, Yiming Du, Ian Godwin, Lingqiang Wang, Peng Lv, Guoquan Liu

**Affiliations:** 1 Institute of Millet Crops, Hebei Academy of Agriculture and Forestry Sciences and Hebei Branch of China National Sorghum Improvement Centre, Shijiazhuang, China; 2 State Key Laboratory of Conservation and Utilization of Subtropical Agricultural Biological Resources, Guangxi Key Laboratory of Sugarcane Biology, College of Agriculture, Guangxi University, Nanning, China; 3 College of Plant Science and Technology, Huazhong Agricultural University, Wuhan, China; 4 Plant Synthetic Biology Australia, School of Agriculture, Food and Wine, Adelaide University, Waite Campus, Glen Osmond, SA, Australia; 5 Hebei Youth Cadres Administrative College, Shijiazhuang, China; 6 Centre for Crop Science, Queensland Alliance for Agriculture and Food Innovation, The University of Queensland, St Lucia, QLD, Australia

**Keywords:** food security, climate change, sorghum, genetic engineering, genome editing, apomixis

The funder “HAAFS International Science and Technology Cooperation Project (2026KJCXZX-GZS-GH01) and Shijiazhuang Foreign Intelligence Introduction Project (20260014)” were erroneously omitted.

The original version of this article has been updated as following

“The author(s) declared that financial support was received for this work and/or its publication. The sorghum project was supported by Central Government Guidance for Local Science and Technology Development Funds (No. 246Z2909G), the Guangxi Science and Technology Support Initiative (Guike AD25069107), the China Agriculture Research System (CARS-06-14.5-B5), National Key Research and Development Program of China (No. 2023YFD1902600), and open funds of the State Key Laboratory of Plant Environmental Resilience (SKLPERKF202511). The research was also funded by the Department of Education of Guangxi Zhuang Autonomous Region, HAAFS International Science and Technology Cooperation Project (2026KJCXZX-GZS-GH01), and Shijiazhuang Foreign Intelligence Introduction Project (20260014). This work was supported by the Super sorghum project UOQ2510-003RTX funded by Grains Research and Development Corporation (GRDC)”.

